# SCOUP: a probabilistic model based on the Ornstein–Uhlenbeck process to analyze single-cell expression data during differentiation

**DOI:** 10.1186/s12859-016-1109-3

**Published:** 2016-06-08

**Authors:** Hirotaka Matsumoto, Hisanori Kiryu

**Affiliations:** Bioinformatics Research Unit, Advanced Center for Computing and Communication, RIKEN, 2-1 Hirosawa, Wako, Saitama, 351-0198 Japan; Department of Computational Biology and Medical Sciences, Faculty of Frontier Sciences, The University of Tokyo, 5-1-5 Kashiwanoha, Kashiwa, Chiba, 277-8561 Japan

**Keywords:** Single-cell transcriptomics, Differentiation analysis, Ornstein–Uhlenbeck process

## Abstract

**Background:**

Single-cell technologies make it possible to quantify the comprehensive states of individual cells, and have the power to shed light on cellular differentiation in particular. Although several methods have been developed to fully analyze the single-cell expression data, there is still room for improvement in the analysis of differentiation.

**Results:**

In this paper, we propose a novel method SCOUP to elucidate differentiation process. Unlike previous dimension reduction-based approaches, SCOUP describes the dynamics of gene expression throughout differentiation directly, including the degree of differentiation of a cell (in pseudo-time) and cell fate. SCOUP is superior to previous methods with respect to pseudo-time estimation, especially for single-cell RNA-seq. SCOUP also successfully estimates cell lineage more accurately than previous method, especially for cells at an early stage of bifurcation. In addition, SCOUP can be applied to various downstream analyses. As an example, we propose a novel correlation calculation method for elucidating regulatory relationships among genes. We apply this method to a single-cell RNA-seq data and detect a candidate of key regulator for differentiation and clusters in a correlation network which are not detected with conventional correlation analysis.

**Conclusions:**

We develop a stochastic process-based method SCOUP to analyze single-cell expression data throughout differentiation. SCOUP can estimate pseudo-time and cell lineage more accurately than previous methods. We also propose a novel correlation calculation method based on SCOUP. SCOUP is a promising approach for further single-cell analysis and available at https://github.com/hmatsu1226/SCOUP.

**Electronic supplementary material:**

The online version of this article (doi:10.1186/s12859-016-1109-3) contains supplementary material, which is available to authorized users.

## Background

Conventional analyses of bulk cells, such as bulk transcriptome analyses, are based on the averaged data of an ensemble of cells and cannot reveal the states of individual cells. Therefore, such analyses cannot distinguish cell types due to the effect of averaging across all cells in a sample, unless each cell lineage is divided in advance by using prior knowledge, such as marker genes. Additionally, bulk transcriptome during differentiation is usually the ensemble of the cells of different degrees of differentiation and information regarding changes in cellular state is smeared. Accordingly, the accurate investigation for gene expression dynamics and regulatory relationships among genes during differentiation are difficult.

With the advent of single-cell technologies, such as single-cell RNA-seq, quantification of the comprehensive states of individual cells is possible [1]. Using single-cell technologies, investigations of cellular states and its transition processes, such as the classification and identification of cell types [2–4], reconstruction of cell lineages [5, 6], and embryonic development [7, 8], have made remarkable progress. Single-cell data is also useful for elucidating cell fate decision mechanisms of multi-lineage differentiation from a single progenitor cell type [9, 10]. Thus, single-cell technologies have the power to shed light on differentiation in particular [11, 12].

To fully analyze the single-cell expression data during differentiation, novel computational methods are necessary [11, 13]. First, ordering of the cells based on expression data so that the order represents the trajectory of differentiation is necessary to investigate gene expression dynamics and regulatory mechanisms. Although experimental time can be used for ordering cells, even cells derived from the same time-point can exhibit different degrees of differentiation [14]. Moreover, computational ordering method is often useful to reconstruct the differentiation process from in vivo snap-shot data, which contains cells at distinct stages of differentiation [5]. Second, estimating the lineage of the cells is necessary to investigate multi-lineage differentiation. Although the expression of marker genes will be useful to classify cell lineages, the prior knowledge of marker genes is limited. Therefore, a lineage estimation method without prior knowledge is necessary to fully analyze the mechanisms of cell fate decisions.

Wanderlust [15] is a pioneering study for ordering cells based on expression data. It uses N-dimensional space composed by N marker genes and constructs the I-nearest neighbor graph in the space, and then reconstructs the differentiation path based on the graph. The degree of differentiation of a cell (in pseudo-time) is defined by the position on the path. Although Wanderlust is a promising method for reconstructing the differentiation path, it will not work when prior knowledge of marker genes is not given. Therefore, several methods that do not require the prior knowledge of marker genes have been developed to order cells [14, 16, 17]. These methods use dimension reduction techniques, such as principal component analysis (PCA), and reconstruct the differentiation path in reduced space using several approaches, such as minimum spanning tree (MST) and principal curves. Each cell is projected onto the reconstructed path and pseudo-time is defined by the projected position on the path. To estimate cell lineage from expression data, a few methods, which use the same framework, have been developed. Monocle [14], a dimension reduction-based approach, estimates the lineage of each cell by estimating multiple paths in reduced space and assigning each cell to one of the paths. These approaches are powerful tools to reconstruct the differentiation process without prior knowledge, and the development of such computational methods will help reveal the mechanisms of differentiation in conjunction with the advancement of single-cell technologies.

However, pseudo-time estimation and cell lineage estimation based on dimension reduction have several problems. For example, interpreting the biological meaning of the path in reduced space is difficult. Additionally, the position in reduced space is affected by noise and gene expression that is irrelevant to differentiation, and the results can therefore change significantly in a subsequent analysis. Moreover, deterministic approaches, such as applications of MST in reduced space, cannot quantify the subtle differences among cells and are inadequate to estimate the lineages of cells at an early stage of bifurcation, which are important for analyzing cell fate decisions. Hence, we developed another approach based on stochastic processes.

In this research, we developed a novel method SCOUP (a probabilistic model to analyze Single-Cell expression data during differentiation with Ornstein–Uhlenbeck Process). SCOUP describes the dynamics of gene expression throughout differentiation directly, including pseudo-time and cell fate of individual cells. SCOUP is based on the Ornstein–Uhlenbeck (OU) process, which represents a variable moving toward an attractor with Brownian motion. In the case of differentiation, an attractor is regarded as a stable expression pattern of a gene after differentiation, and hence, an OU process is appropriate to describe expression dynamics throughout differentiation. Because OU processes suppose only a single attractor and cannot represent multi-lineage differentiation, we expand the typical OU process into a mixture OU process by representing the cell fate of each cell and lineage-specific expression patterns with latent values and different attractors, respectively. We compared the accuracy of pseudo-time estimates from SCOUP with those of previous methods using time-series scqPCR and scRNA-seq, and SCOUP was superior to previous methods in almost all conditions. We also evaluated the cell lineage estimation using scqPCR data in which cells exhibit multi-lineage differentiation. SCOUP successfully estimated cell lineage more accurately than Monocle, especially for cells at an early stage of bifurcation. In addition, SCOUP represents each gene expression dynamic directly and can be applied to various downstream analyses. As an example, we developed a novel correlation calculation method for elucidating regulatory relationships among genes. We normalized data based on the optimized parameters in our model, which assumes the conditional independency among genes, and calculated correlations within normalized data, and this method detected covariance that cannot be explained by the model alone. We applied this method to scRNA-seq data and detected a candidate of key regulator for differentiation and clusters in a correlation network which were not detected with conventional correlation analysis.

We proposed a novel theoretical and computational method SCOUP to analyze single-cell data. The theoretical basis of SCOUP will be useful not only for pseudo-time and cell lineage estimation, but also for various biological analyses such as gene regulatory network inference. In particular, SCOUP can represent continuous-time stochastic dynamics and is suited for analyzing time-series data. As the number of single-cell data with high temporal resolution is increasing, computational methods for analyzing such data are becoming more important. Thus, SCOUP is a promising approach for further single-cell analysis and bioinformatics method development.

## Methods

### Ornstein-Uhlenbeck process

Let *X*_*t*_ be an OU process. *X*_*t*_ satisfies the following stochastic differentiation equation:

1 $$\begin{array}{*{20}l} {dX}_{t} = -\alpha\left(X_{t} - \theta\right)dt + \sigma {dW}_{t}, \end{array} $$

where *α*, *θ*, *σ*, and *W*_*t*_ denote the strength of relaxation toward the attractor, the value of the attractor, the strength of noise, and “white noise,” respectively. If the initial value is given by *X*_0_, the value at time *t* (*X*_*t*_) satisfies the following normal distribution:

2 $$\begin{array}{*{20}l}{} &P\!\left(X_{t}|X_{0}, \alpha, \sigma^{2}, \theta, t\right) \,=\, \mathcal{N}\! \left(X_{t}|\mathrm{e}^{-\alpha t}X_{0}\,+\,\left(1\!-\mathrm{\!e}^{-\alpha t}\right)\theta, \frac{\sigma^{2}(1\,-\,\mathrm{e}^{-2\alpha t})}{2\alpha} \!\right). \end{array} $$

**Fig. 1:**
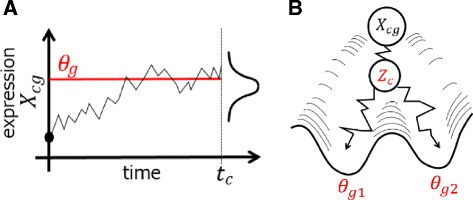
The conceptual diagrams of the OU process (**a**) and SCOUP for multi-lineage differentiation (**b**). **a** The OU process represents a variable (i.e., expression of a gene *g* in a cell *c*) moving toward attractor (*θ*_*g*_) with Brownian motion. The value at time *t* satisfies the normal distribution (see “Methods”). **b** Each lineage has distinct attractor (*θ*_*g*1_ and *θ*_*g*2_), and the lineage of a cell *c* is represented with latent value *Z*_*c*_. The expression of gene *g* in cell *c* is described with the mixture OU process

This OU process represents a variable moving toward attractor *θ* with Brownian motion (Fig. 1a) and has been used to describe adaptive evolution of a quantitative trait along phylogenetic tree [18], for example.

In the process of cellular differentiation, a cell changes from one cell type to another, and its expression pattern changes from a specific pattern to a different specific pattern. Moreover, each single cell exhibits different degrees of differentiation, and therefore a continuous-time model is necessary to represent single-cell expression dynamics. With the OU process, we can describe such dynamics by considering that *X*_0_ and *θ* are the expression patterns of progenitor cells and differentiated cells, respectively. In addition, other parameters *α* and *σ* can be regarded as the speed of expression change and level of noise, respectively. Thus, the OU process is suitable for modeling gene expression dynamics throughout differentiation. In this research, we extended the OU process for single-cell expression data and developed a parameter optimization method.

### OU process for single lineage differentiation

We developed a probabilistic model for single lineage differentiation. Hereinafter, we denote the number of cells, the number of genes, the cell index, and the gene index as *C*, *G*, *c*, and *g*, respectively. We assume that expression in each cell is independent and that the total probability *P*(*E*|*Φ*,*T*), where *E* is the expression data of all cells and genes and *Φ* is the set of parameters, is the product of cell probabilities. Each cell has a degree of differentiation progression parameter (i.e., pseudo-time) *t*_*c*_. Although genes interact with each other and multivariate OU process can be more appropriate to describe all gene expression dynamics, multivariate OU process requires more computational and analytical complexity. Therefore, we assume that each gene follows its OU process independently and has parameters *α*_*g*_, ${\sigma _{g}^{2}}$, and *θ*_*g*_. Despite the above assumption, we can infer the regulatory relationship between genes by calculating the covariance that is not explained by gene independent model (as explained in the section on “Correlation between genes”). Thus, a cell probability is the product of gene expression probability *P*(*E*_*cg*_|*Φ*_*g*_,*t*_*c*_), where *E*_*cg*_ is the expression data of gene *g* in cell *c*. Thus, the probability of single lineage differentiation is given by

3 $$ \begin{aligned} P(E|\Phi,T) &= \prod_{c=1}^{C} \prod_{g=1}^{G} P(E_{cg}|\Phi_{g},t_{c})\\ &= \prod_{c=1}^{C}\prod_{g=1}^{G} \int {dS}_{cg} P_{\text{ou}}(E_{cg}|S_{cg}, \Phi_{g}, t_{c})P(S_{cg}), \end{aligned}  $$

where $\Phi _{g}=(\alpha _{g}, {\sigma _{g}^{2}}, \theta _{g})$, *Φ*={*Φ*_*g*_|*g*=1,…,*G*}, *T*={*t*_*c*_|*c*=1,…,*C*}, *S*_*cg*_ is the expression of gene *g* in cell *c* at *t*=0, and *P*_ou_ is a probability distribution based on an OU process and described by the following normal distribution:

4 $${} \begin{aligned} &P_{\text{ou}}\left(E_{cg}|S_{cg}, \Phi_{g}, t_{c}\right)\\ &= \mathcal{N} \left(E_{cg}|\mathrm{e}^{-\alpha_{g} t_{c}}S_{cg}\,+\,\left(1-\mathrm{e}^{-\alpha_{g} t_{c}}\right)\theta_{g}, \frac{{\sigma_{g}^{2}}\left(1-\mathrm{e}^{-2\alpha_{g} t_{c}}\right)}{2\alpha_{g}} \right). \end{aligned}  $$

*P*(*S*_*cg*_) is the initial distribution of a gene and is given by a normal distribution as follows:

5 $$\begin{array}{*{20}l} P(S_{cg}) &= \mathcal{N}\left(S_{cg}|\mu_{0g}, \sigma_{0g}^{2}\right). \end{array} $$

Although optimization of these initial parameters is possible, a fully differentiated state may be regarded as an initial state and pseudo-time may be inferred in the reverse order of differentiation. In this way, deciding the direction of differentiation without the knowledge of initial condition is difficult. Moreover, the expression data of progenitor cells are available in many experimental studies. Therefore, we assume that *μ*_0*g*_ and $\sigma _{0g}^{2}$ are known in this research.

### Sufficient statistic for OU processes

Like a continuous Markov model for nucleotide evolution [19], the continuous OU process can be regarded as the limit of a discrete time OU process. *P*_ou_(*E*_*cg*_|*S*_*cg*_,*Φ*_*g*_,*t*_*c*_) can be described as follows:

6 $${} P_{\text{ou}}\left(E_{cg}|S_{cg},\Phi_{g}, t_{c}\right)={\lim}_{N\rightarrow \infty} P_{N}\left(X_{cgN}|X_{cg0},\Phi_{g}, t_{c}\right)  $$

7 $${} P_{N}\!\left(X_{cgN}|X_{cg0},\!\Phi_{g}, t_{c}\right)\,=\,\int \!\!{dX}_{cg} \!\prod_{s=1}^{N} \!P_{\text{ou}}\!\left(X_{cgs}|X_{cgs-1}, \!\Phi_{g}, t_{c}/\!N\right)  $$

8 $${} P\left(X_{cg}|\Phi_{g}, t_{c}\right)= \prod_{s=1}^{N} P_{\text{ou}}\left(X_{cgs}|X_{cgs-1}, \Phi_{g}, t_{c}/N\right) P(X_{cg0}),  $$

where *X*_*cg*_={*X*_*cgs*_|*s*=0,…,*N*} represents a path such that *X*_*cg*0_ and *X*_*cgN*_ satifsy *S*_*cg*_ and *E*_*cg*_, respectively. In other words, *X*_*cgs*_ corresponds to the variable at time *st*_*c*_/*N*. In this model, we assume *S*_*cg*0_ is fixed and consider *X*_*cg*_ as *X*_*cg*_∈{*X*_*cgs*_|*s*=1,…,*N*} for simplicity (see Additional file 1 for the calculations related to *S*_*cg*0_). Accordingly, we consider the likelihood of *X*_*cg*_ as follows:

9 $$\begin{array}{*{20}l} P\left(X_{cg}|S_{cg},\Phi_{g}, t_{c}\right)&= \prod_{s=1}^{N} P_{\text{ou}}\left(X_{cgs}|X_{cgs-1}, \Phi_{g}, t_{c}/N\right). \end{array} $$

According to the expansion of the above likelihood, the log-likelihood of *X*_*cg*_ is described as follows (see Additional file 1 for detailed calculation). Here, we abbreviate the indexes *c* and *g* and represent *X*_*cg*_ and *X*_*cgs*_ as *X* and *X*_*s*_ for simplicity.

10 $$ \begin{aligned}{} l(X) =& \sum_{s=1}^{N} \ln P_{\text{ou}}\left(X_{s}|X_{s-1}, \Phi_{g}, t/N\right)\\[-2pt] =& -\frac{N}{2}\ln\frac{\alpha}{\pi\sigma^{2}\left(1-\mathrm{e}^{-2\alpha t}\right)}\\[-2pt] & -\frac{N}{2t\sigma^{2}}\left(2\left(\sum_{s=1}^{N-1}{X_{s}^{2}} - \sum_{s=0}^{N-1}X_{s}X_{s+1}\right)+ {X_{0}^{2}} + {X_{N}^{2}}\right)\\[-2pt] & + \frac{\alpha}{2\sigma^{2}}\left({X_{0}^{2}}-{X_{N}^{2}}- 2\theta X_{0} + 2\theta X_{N}\right)\\[-2pt] & +\!\frac{\alpha^{2} t}{2N\sigma^{2}}\!\!\left(\!-2\!\sum_{s=1}^{N-1}\!{X_{s}^{2}}\,+\,\sum_{s=0}^{N-1}\!X_{s}X_{s+1}\,+\,2\theta\!\sum_{s=1}^{N-1}\!X_{s} \,-\,N\theta^{2}\right)\\ & + \mathcal{O}(1/N). \end{aligned}  $$

Accordingly, we can calculate the log-likelihood by using the following statistics $\sum _{s=1}^{N-1}{X_{s}^{2}}$, $\sum _{s=0}^{N-1}X_{s}X_{s+1}$, and $\sum _{s=1}^{N-1}X_{s}$.

The expected values of the above statistics are sufficient for parameter optimization. The posterior probability *P*(*X*_1_…*X*_*N*−1_|*X*_*N*_,*X*_0_) is regarded as the multivariate normal distribution, and the expectation of *X*_*s*_ and ${X_{s}^{2}}$ can be calculated from the mean and variance–covariance matrix of the multivariate normal distribution. However, the expansion of the posterior probability gives only the (*N*−1)×(*N*−1) precision matrix, and we must therefore calculate the inverse of the matrix to obtain the variance–covariance matrix. Although we cannot use numerical methods to solve the inverse of the precision matrix because we consider *N* as the limit for infinite, we can solve for the inverse matrix analytically by using the tridiagonal property of the precision matrix [20]. By hand calculation, we showed that the expected values of these statistics were able to be solved analytically. For example, the expected value of one of the statistics is as follows:

11 $$\begin{array}{*{20}l}{} \sum_{s=1}^{N-1}\!<X_{s}>=\! \frac{X_{0}+X_{N}-2\theta}{\sinh \alpha t} \sum_{s=1}^{N-1}\sinh \left(s\frac{\alpha t}{N}\right) \,+\, (N\,-\,1)\theta \,+\, \mathcal{O}(1/N). \end{array} $$

The detailed calculation is described in the Additional file 1.

### EM algorithm

We employed a parameter optimization using an expectation–maximization (EM) algorithm. When the likelihood function contains unobserved variables, an EM algorithm can be used for parameter optimization. The EM algorithm runs E step and M step iteratively and finds parameters that satisfy the local maximum of the marginal likelihood function. In the E step, we calculate the expectation of a specific statistic with current parameters. In the M step, we calculate the expected log-likelihood function (Q function) and optimize parameters so that they maximize the Q function. In our model, the path *X*_*cg*1_…*X*_*cgN*−1_ is unobserved, and the Q function is as follows:

12 $$ \begin{aligned} {} &\mathcal{Q}\left((\Phi,T),\left(\Phi^{\text{old}},T^{\text{old}}\right)\right)\\ {} &=\!\! \prod_{c}\! \prod_{g}\! \int \!\!{dX}_{cg1:N\!-1} P\!\left(\!X_{cg1:N-1}\!|X_{cgN},X_{cg0}, \!\Phi_{g}^{\text{old}},t_{c}^{\text{old}}\right) \!l(X_{cg}), \end{aligned}  $$

where *X*_*cg*1:*N*−1_=(*X*_*cg*1_,*X*_*cg*2_,…,*X*_*cgN*−1_).

The Q function can be expanded analytically with an expected value of the statistic described in the previous section. Thus, we can optimize *Φ*_*g*_ by solving $d\mathcal {Q}/d\theta _{g}=0,d\mathcal {Q}/d\alpha _{g}=0,d\mathcal {Q}/d{\sigma _{g}^{2}}=0$, which results in the following equations:

13 $${} \theta_{g}^{*} = \theta_{g} + \frac{1}{\sum t_{c}}\sum_{c}\frac{2 \left(X_{cgN}-\mathrm{e}^{-\alpha_{g} t_{c}}X_{cg0} -\left(1-\mathrm{e}^{-\alpha_{g} t_{c}}\right)\theta_{g}\right)}{\alpha_{g} \left(1+\mathrm{e}^{-2\alpha_{g} t_{c}}\right)}  $$

14 $${} \alpha_{g}^{*} = \frac{\sum_{c}\left(-t_{c}{\sigma_{g}^{2}} - \left(X_{cg0}-\theta_{g}\right)^{2}+\left(X_{cgN}-\theta_{g}\right)^{2}\right)}{\sum_{c} Z^{\alpha_{g}}_{c}}  $$

15 $${} \sigma_{g}^{*2} \!= \!\frac{1}{C}\!\sum_{c} \!\frac{2\alpha_{g}}{1\,-\,\mathrm{e}^{-2\alpha_{g} t_{c}}}\!\left(X_{cgN} \,-\, \mathrm{e}^{-\alpha_{g} t_{c}}\!X_{cg0} \,-\, \left(\!1\,-\,\mathrm{e}^{-\alpha_{g} t_{c}}\right)\!\theta_{g} \right)^{2},  $$

where *Z*^*α*^ is explained in the Additional file 1. The pseudo-time variable *t*_*c*_ cannot be optimized analytically, and we therefore solve *t*_*c*_ to satisfy $d\mathcal {Q}/{dt}_{c}=0$ by Newton’s method.

In cases, *X*_*cg*0_ is also unobserved, so we must calculate the expected value of *X*_*cg*0_. As such, we calculate the expected values, including the expected value of *X*_*cg*0_ and $X_{cg0}^{2}$, in the E step and optimize parameters with the above equation in the M step. The detailed optimization process and calculation are described in the Additional file 1.

We validated our parameter optimization method with simulation data and confirmed that SCOUP succeeded to optimize parameters so that the marginal likelihood was maximized (see Additional file 1).

### Mixture OU process for multi-lineage differentiation

We also extended the single lineage model to a mixture model in order to consider multi-lineage differentiation, such as bifurcation (Fig. 1b). We assume that the number of lineages is known and given by *K* and that each lineage has a different attractor *θ*_*gk*_. The fate of a cell *c* is unknown and is represented with the latent value *Z*_*c*_, which is 1 of *K* representations. With this latent value, the mixture OU process is given by

16 $${} P\left(E_{c},S_{c}\right) = \sum_{k=1}^{K} \pi_{k} \prod_{g=1}^{G} P_{\text{ou}}\left(E_{cg}|S_{cg}\alpha_{g}, \sigma_{g}, \theta_{gk}, t_{c}\right)P(S_{cg})  $$

17 $${} P\!\left(E_{c},S_{c},Z_{c}\right) \,=\,\! \prod_{k=1}^{K} \!\pi_{k}^{Z_{ck}} \!\prod_{g=1}^{G}\! \left(P_{\text{ou}}\!\left(E_{cg}|\alpha_{g}, \sigma_{g}, \theta_{gk}, t_{c}\right)P(S_{cg})\!\right)^{Z_{ck}} \!,  $$

where *π*_*k*_ is the probability of lineage *k*. This mixture model describes the multi-lineage case that each lineage diverges from the common initial distribution (one-step bifurcation model). This mixture model is a basic model for describing bifurcation and will be a useful method to analyze several bifurcation processes. Even in the cases that progenitor cells differentiate into different lineages through multi-step bifurcation, we can use the same model to represent multi-step processes by combining the one-step bifurcation models. However, the OU process with multi-step bifurcation becomes mathematically difficult and we leave it for future work.

Here, *Z*_*c*_ is an unobserved value, and we maximize the marginal likelihood with the EM algorithm. As described in the previous section, we must calculate the expectation of the unobserved value to calculate the Q function. The posterior probability of *Z*_*c*_ and the expectation of *Z*_*c*_ (*γ*_*ck*_) are described as follows:

18 $$\begin{array}{*{20}l} &P(Z_{c}|E_{{cg}},S_{cg},) \propto \prod_{k=1}^{K}\left(\pi_{k}^{Z_{{ck}}}\prod_{g=1}^{G} P_{\text{ou}}(E_{cg}|S_{cg}, \theta_{{gk}}, t_{c})^{Z_{{ck}}}\right) \end{array} $$

19 $$\begin{array}{*{20}l} &\gamma_{{ck}} = E[Z_{{ck}}] = \frac{\pi_{k}\prod_{g=1}^{G} P_{\text{ou}}(E_{{cg}}|S_{{cg}}, \theta_{{gk}}, t_{c})}{\sum_{k'} \pi_{k'}\prod_{g=1}^{G} P_{\text{ou}}(E_{{cg}}|S_{{cg}}, \theta_{gk'}, t_{c})}. \end{array} $$

By using the above equation and previous description, we can calculate the Q function analytically. We optimize

20 $$\begin{array}{*{20}l} \pi_{k} &= \frac{\sum_{c} \gamma_{ck}}{\sum_{c}\sum_{k'} \gamma_{ck"}} \end{array} $$

by solving *dQ*/*dπ*_*k*_=0. Other parameters are optimized likewise using the single lineage model. Accordingly, we calculate the expected values of variables, such as *γ*_*ck*_ and *S*_*cg*0_, in the E step and update parameters in the M step.

The lineage of a cell is estimated from the expectation of the latent value of a cell (*γ*_*c*_). SCOUP can quantify the certainty of the estimated lineage of a cell from the the value of *γ*_*c*_.

### Initialization of time parameter

Our method might converge to undesirable local optima if *T* is initialized randomly. For example, estimated pseudo-time might be inferred in the reverse order of differentiation. To avoid undesirable local optima, rough initialization of *T* is effective. Although experimental time will be useful for initialization, such data are not always available. For example, experimental time does not exist for expression data of an in vivo snap-shot sample [5]. Therefore, an initialization method that does not depend on experimental time is necessary. Here, we explain our initialization method based on a dimension reduction approach.

we developed dimension reduction approach for pseudo-time initialization, called SP (pseudo-time calculation based on Shortest Path from the root cell in the MST). Firstly, we added the mean of the initial distribution (*μ*∈{*μ*_*g*0_|*g*=1…*G*}) to expression data and regarded it as an initial point for the pseudo-time calculation. Next, we performed PCA, constructed MSTs in the reduced space, searched for the shortest path from an initial point using Prim’s algorithm, and regarded the weight of the shortest path as the pseudo-time. In this paper, we set the dimensionality of the PCA to two and used this pseudo-time for the initialization of our method.

### Dimension reduction approach

In this section, we explain the previous pseudo-time estimation methods based on a dimension reduction approach.

Monocle [14] constructs a MST in reduced space, searches for the longest path in the MST, and estimates pseudo-time along the longest path. We added the mean of the initial distribution data and regarded it as an initial point for the pseudo-time calculation. We used all genes in a dataset as marker genes and the other parameters of Monocle were set to default values, unless otherwise specified.

TSCAN [17] performs model-based clustering in reduced space, connects clusters, and estimates pseudo-time by projecting cells onto the connected path. Although TSCAN can infer an order of clusters, it cannot regard a point as an initial point. Therefore, we compared the accuracy of outputted pseudo-time with reversed pseudo-time and defined the pseudo-time of TSCAN as the superior one. Because TSCAN failed to output pseudo-time of partial cells when we set a high number of clusters, we set the number of clusters to three in this research.

In this paper, we compared the performance of SCOUP with those of above dimension reduction-based methods in addition to SP. Although Wanderlust is also a useful method to estimate pseudo-time and cell lineage, we exclude it from comparison. This is because we consider the condition that the prior knowledge of marker genes is not given and Wanderlust is designed not for single-cell qPCR and RNA-Seq but for mass and flow cytometry data.

### Correlation between genes

We also proposed a novel correlation function between two genes. Although we assume the conditional independence among genes to represent gene dynamics, we can detect the regulatory relationship between genes by calculating the covariance. Our correlation function quantifies the covariance between genes that is not explained by our model.

For time-series data, a ordinal correlation coefficient will be high even if two variables only have similar time-trend. For example, any two independent genes that are upregulated in accordance with differentiation exhibit a high correlation. In the case of the detection of interactions between genes, it is most appropriate to remove the influence of time-trend. To remove this trend effect, the expression data at a specific experimental time point is often used to calculate the correlation. However, this approach is insufficient to remove the time effect resulting from the difference between the experiment time and the progression of cells. Accordingly, the trend effect is best removed by using cells within a specific pseudo-time span for calculation. Although this analysis will remove the trend effect, the number of cells that are used for the calculation decreases owing to the limit of the span of pseudo-time and precise calculation will therefore be difficult.

Several methods have been developed to calculate correlation while removing the confounding effects. For example, scLVM [2] revealed hidden subpopulations from single-cell RNA-seq data by removing the effects, such as cell cycle. In this research, we developed a novel correlation function based on our probabilistic model to remove the effect of time-trend. As described in the section on “OU process for single lineage differetiatiation” and the Additional file 1, the probabilistic distribution of the expression of a gene *g* at time *t* (*X*_*tg*_) is described as follows:

21 $$\begin{array}{*{20}l}{} P\left(X_{tg}|\Phi_{g},t_{c}\right) &= \int {dS}_{g} P_{\text{ou}}\left(X_{tg}|S_{g}, \Phi_{g}, t\right)P(S_{g})= \mathcal{N}\left(X_{tg}|\mu_{tg}, \sigma^{2}_{tg}\right), \end{array} $$

where

22 $$\begin{array}{*{20}l} \mu_{tg} &= \mathrm{e}^{-\alpha_{g} t}\mu_{0g} + \left(1-\mathrm{e}^{-\alpha_{g} t}\right)\theta_{g} \end{array} $$

23 $$\begin{array}{*{20}l} \sigma^{2}_{tg} &= \frac{{\sigma_{g}^{2}}\left(1-\mathrm{e}^{-2\alpha_{g} t}\right)}{2\alpha_{g}} + \mathrm{e}^{-2\alpha_{g} t}\sigma_{0g}^{2}. \end{array} $$

As such, we can remove the time dependency by standardizing the time-dependent mean and variance as follows:

24 $$\begin{array}{*{20}l} Z_{cg} &= \frac{E_{cg} - \mu_{t_{c}g}}{\sigma^{2}_{t_{c}g}}. \end{array} $$

We calculated the correlation coefficient for the above standardized values. This correlation function can detect gene pairs that exhibit interactions that are unexplained by the model, which assume the conditional independence among genes.

The above standardization assumes a single normal distribution and is not suitable for multi-lineage model. However, max*kγ*_*ck*_ of most cells, which we analyzed, were about 1.0, and hence, most cells would be assigned to one of the lineage. Therefore, the standardization will be effective by assigning a cell to a relevant lineage. In addition, correlation of each lineage will be calculated by dividing cells into each lineage in advance.

### Dataset

#### Single-cell qPCR for single-lineage differentiation

We used the time-series single-cell qPCR dataset produced by Kouno’s group [21] from THP-1 human myeloid monocytic leukemia cells differentiating into macrophages. They investigated the expression of 45 transcription factors by 120 single cells at each eight time point (0, 1, 6, 12, 24, 48, 72, and 96 h) after phorbol myristate acetate stimulation.

To evaluate the estimated pseudo-time in many conditions, we constructed a dataset, (Kouno’s data (1)) follows. We added noise to raw expression data as described below to investigate the effect of noise in pseudo-time estimation. We added noise to raw expression data *E*_*cg*_ by adding $\bar E_{g}\times U_{R}[0,\epsilon ]$, where $\bar E_{g}$ is the mean expression of a gene and *U*_*R*_[0,*ε*] is a uniform random number from 0 to *ε*. We produced 20 replicates for each *ε* (noise level), and validated the pseudo-time of each method for each noise level.

We also constructed another dataset, (Kouno’s data (2)), to validate lineage estimation by adding 45 pseudogenes that exhibit various expression patterns among lineages. We initially selected 60 cells randomly from 120 cells at a given time point. The expression $E_{cg'}\phantom {\dot {i}\!}$ of a pseudogene *g*^′^ by the selected cells is equal to raw expression ($\phantom {\dot {i}\!}E_{cg'} = E_{cg}$). For the remaining cells, we inverted the raw expression in relation to the initial mean ($\phantom {\dot {i}\!}E_{cg'} = -2E_{cg} + \mu _{0g}$). We also added noise as mentioned above in regard to Kouno’s data (1). Because Monocle cannot accept negative values, we incremented the values by a minimum of 1 to make the expression positive.

The initial distribution $\left (\mu _{0g}~\text {and}~\sigma _{0g}^{2}\right)$ was calculated from 0-h cells as follows,

25 $$\begin{array}{*{20}l} \mu_{0g} &= \frac{\sum_{c\in{C_{0}}} E_{cg}}{|C_{0}|}, \end{array} $$

26 $$\begin{array}{*{20}l}[-2pt] \sigma_{0g}^{2} &= \frac{\sum_{c\in{C_{0}}} \left(E_{cg} - \mu_{0g}\right)^{2}}{|C_{0}|}, \end{array} $$

where *C*_0_ is the set of 0-h cells and |*C*_0_| is the number of 0-h cells.

#### Single cell qPCR for bifurcation

To validate the lineage estimation in real data, we used a dataset produced by Moignard’s group [22]. They investigated the single-cell qPCR results for 46 transcription factors throughout hematopoietic development from embryonic day (E) 7.0 to E8.5 in mouse embryos. These data include a lineage bifurcation between E7.75 and E8.25; at this time, head fold (HF) cells differentiate into putative blood and endothelial populations, which are distinguished as either GFP^+^ cells (4SG) or Flk1^+^GFP^−^ cells (4SFG^−^). We used the expression profiles of HF, 4SG, and 4SFG^−^ and investigated whether SCOUP and Monocle can classify 4SG and 4SFG^−^ using only their expression profiles. We randomly selected 1000 cells because Monocle did not seem to work correctly for a large number of cells and this procedures left 364 HF cells, 360 4SG cells, and 276 4SFG^−^ cells. The initial distribution was calculated from HF cells in the same way as Kouno’s data.

#### Single-cell RNA-seq for single-lineage differentiation

We also investigated the stimulation time-series single-cell RNA-seq dataset (at 0, 1, 4, and 6 h) for primary mouse bone-marrow-derived dendritic cells that was produced by Shalek’s group [23]. This dataset contains data for three different time series corresponding to each of the different stimulation methods: lipopolysaccharide (LPS), viral-like double-stranded RNA (PIC), and synthetic mimic of a bacterial lipopeptides (PAM). First, we converted transcripts per million (TPM) to log(TPM+1) and defined this value as gene expression. Next, we removed outlier cells so that each cell in the dataset contained more than 4000 genes with detectable levels of expression; this left 281 LPS cells, 224 PAM cells, and 159 PIC cells. Third, we calculated the absolute difference in mean gene expression between the 1-h cells and 6-h cells for each stimulation. We extracted the top 1000 genes in descending order of this difference for each stimulation and used these genes for pseudo-time estimation. We also added unstimulated cells (outlier cells were removed through a procedure like that described above, leaving 85 cells) to the LPS, PAM, and PIC data and regarded these cells as 0-h data. The initial distribution was calculated from unstimulated cells in the same way as Kouno’s data.

### Accuracy measure

#### Pseudo-time evaluation

To evaluate the accuracy of pseudo-time estimated from each method, we regarded experimental time as genuine time and calculated the rate of inconsistency between pseudo-time and experimental time. By using the accuracy measure of TSCAN as a reference, we evaluated the inconsistency by calculating the rate of cell pairs whose pseudo-time ordering was inconsistent with experimental-time ordering, and we defined the pseudo-time inconsistency score (PIS) as follows:

27 $$\begin{array}{*{20}l} \text{PIS} &= \frac{\sum_{(i,j) \in \left(t_{i}^{\text{(e)}} < t_{j}^{\text{(e)}}\right)} I(t_{i} > t_{j}) }{\sum_{(i,j) \in \left(t_{i}^{\text{(e)}} < t_{j}^{\text{(e)}}\right)} \left(I(t_{i} < t_{j}) + I(t_{i} > t_{j}) \right)}, \end{array} $$

where $t_{c}^{\text {(e)}}$ and *t*_*c*_ are respectively the experimental time and pseudo-time of cell *c*. *I*(*t*_*i*_<*t*_*j*_) is an indicator function that takes the value 1 if the conditional expression is true.

#### Lineage evaluation

We evaluated the performance of lineage estimation by SCOUP and Monocle by comparing the cell lineage annotation of each cell. The annotation of a cell from simulation data is obvious and that of Moignard’s data is given by 4SG or 4SFG^−^ in accordance with GFP^+^ or Flk1^+^GFP^−^. SCOUP estimates a cell lineage based on the expectation of the posterior probability of cell fate (*γ*_*ck*_). We classified cells into one of two lineages on the basis of whether *γ*_*ck*_ exceeded a threshold. We calculated the precision and recall for each threshold and calculated the area under the curve (AUC) value. Monocle also can estimate cell lineage by setting the parameter *num_paths* to 2, thereby outputting the state of a cell as either state1 (pre-bifurcation), state2 (one lineage), or state3 (another lineage). Monocle is a deterministic method and cannot distinguish subtle differences. Therefore, we regard that state1, state2, and state3 belong to one lineage with probabilities 0.5, 1.0, and 0.0, respectively. We calculated the AUC value for Monocle in the same way.

## Results and discussion

### Validation of parameter optimization

We validated our parameter optimization method with simulation data. We generated simulation data from the normal distribution based on the OU process by varying the parameters. The number of genes and cells are set to 500 and 100, respectively.

**Fig. 2:**
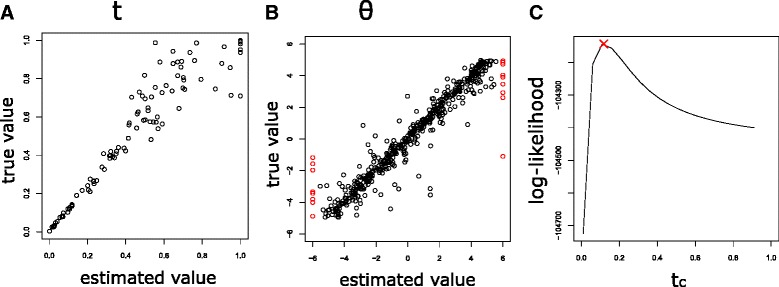
Validation of parameter estimation of SCOUP for simulation data. **a** and **b** is the comparison between the estimated values and true values for pseudo-time (*t*) and *θ*_*g*_, respectively. The outlier whose estimated value exceeds the boundary of drawing area is visualized in the border with a red circle for visualization. **c** is the log-likelihood curve with respect to *t*_*c*_ of a cell. The optimized *t*_*c*_ is indicated with x-max

Firstly, we compared the values of estimated parameters with those of true parameters (Fig. 2a, b). The values of estimated time and estimated *θ*_*g*_ are highly correlated with those of true values (*r*^2^ are 0.94 and 0.96, respectively). The values of estimated mean and variance of the OU process are also highly correlated with those of true mean and variance (0.99 and 0.94, respectively), and hence, SCOUP succeeded to reconstruct the original probabilistic distribution with high accuracy (the details are described in the Additional file 1).

Next, we investigated that the log-likelihood of optimized parameters was higher than those of varied parameters. Figure 2c is the example of the log-likelihood curve with respect to time parameter of a cell (*t*_*c*_), and the value of optimized *t*_*c*_ is drawn with x-mark. The log-likelihood of the optimized *t*_*c*_ was located in the top of the log-likelihood curve. We also verified that the optimized parameters were located in the top of the log-likelihood surface in regards to other parameters (the details are described in the Additional file 1). Thus, SCOUP can optimize the parameters correctly.

### Validation of pseudo-time estimation

In this section, we compared the accuracy of the pseudo-time of each method: SCOUP, our method; SP, pseudo-time estimation based on shortest path in the MST in reduced space; Monocle, dimension reduction-based method that reconstruct differentiation path by the longest path in the MST; TSCAN, dimension reduction-based method that reconstruct differentiation path by running model-based clustering and connecting clusters. For pseudo-time evaluation, we used Kouno’s data (1) and the Shalek’s data.

**Fig. 3:**
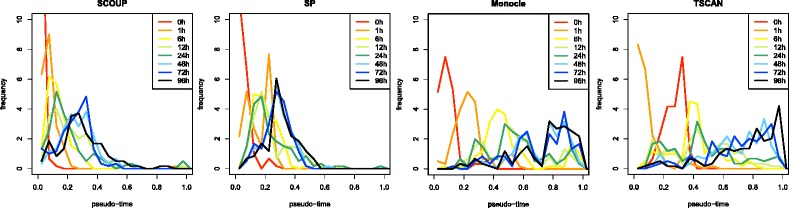
The histograms of pseudo-time estimates produced by each method for Kouno’s data (1) without additional noise. The histograms are drawn for each experimental time point with different colors. The pseudo-time values inferred by SCOUP over 1.0 are integrated into 1.0 for visualization. The pseudo-time values inferred by Monocle and TSCAN are normalized so that maximum = 1.0

Figure 3 shows the histograms of pseudo-time inferred by each method for Kouno’s data (1) without additional noise (*ε*=0). The histograms are drawn for each experimental time point. Although the pseudo-time trends of each method are roughly consistent with experimental time order, each method shows distinctive characteristics. In most cases, the orders of pseudo-time produced by TSCAN for 0-h cells and 1-h cells are reversed. The orders might be reversed in the process of assigning cells to clusters or ordering clusters. In SP, the pseudo-time of the portion of cells is large and that of the remaining cells is relatively small. This is because a portion of the cells must be outliers and are therefore located far from other cells in reduced space. The outliers cause long paths in the MSTs and affect other pseudo-time estimates through normalization. Monocle seems to successfully order cells. In SCOUP, the pseudo-times of 0-h cells are relatively concentrated at *t*=0.0 as compared to the other methods. The pseudo-time of 0-h cells based on dimension reduction approaches is dispersed because 0-h cells tend to scatter in reduced space owing to the dispersion of expression and noise. In contrast, SCOUP contains a noise term in the model and estimates pseudo-time from the trend of total gene expression, which removes the influence of noise. Because 0-h cells are progenitor cells and belong to a steady state before differentiation, it is appropriate to consider the pseudo-time of 0-h cells as approximately 0. Thus, SCOUP successfully identified the initial steady state.

**Fig. 4:**
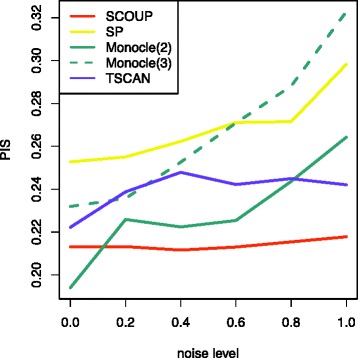
PIS of each method applied to Kouno’s data (1). The *x*-axis represents the noise level (*ε*) (see “Methods”) and the *y*-axis represents the degree of inconsistency between the pseudo-time and experimental time (PIS). Each method is distinguished by color: *red*, SCOUP; *yellow*, SP; *green*, Monocle; and *blue*, TSCAN. We compared the PIS of Monocle for different parameters *m* *a* *x*_*c* *o* *m* *p* *o* *n* *e* *n* *t* *s*, which correspond to dimensions. The solid and dotted lines correspond to *m* *a* *x*_*c* *o* *m* *p* *o* *n* *e* *n* *t* *s*=2 and 3, respectively

Next, we quantitatively evaluated the accuracy of pseudo-time estimated by each method for Kouno’s data (1) based on the pseudo-time inconsistency score (PIS) (Fig. 4). The PISs of SCOUP were superior to those of other methods under most conditions. This demonstrates that SCOUP can estimate pseudo-time well, even from noisy data. Under one condition, the PIS of Monocle was superior to that of SCOUP, and SCOUP was the second best. This can be because SCOUP does not describe the differentiation process completely. For example, SCOUP cannot represent variable attractors, such as transient patterns, and dimension reduction-based methods might be able to accommodate such expression patterns. In future work, we will extend SCOUP to represent such dynamics.

We also investigated the effect of the number of dimensions of reduced space for pseudo-time estimation in Monocle. We set the argument of Monocle *max*_*components*, which corresponds to the number of dimensions, to 2 and 3 and denote Monocle analyses with each configuration as Monocle(2) and Monocle(3), respectively. Across all conditions, Monocle(3) was inferior to Monocle(2). This is because the third dimension of reduced space represents something unrelated to differentiation. Without prior knowledge, it is difficult to set a proper number of dimensions, and pseudo-time can be erroneous under an improper number of dimensions. Although SCOUP is based on a dimension reduction approach in the process of pseudo-time initialization, we verified that the pseudo-time estimated from different numbers of dimensions (i.e., 2 and 3) converged to almost same value in this dataset (*r*^2^=0.94 for *ε*=0.0). Even if the estimated pseudo-times of SCOUP differ, we can infer appropriate pseudo-times by selecting the model with the highest likelihood.

Next, we evaluated the pseudo-time of each method as inferred from Shalek’s data. The PIS of each method is shown in Table 1. Across all conditions, the PISs of SCOUP were superior to those of other methods. Unlike qPCR, RNA-seq provides comprehensive gene expression profiles and contains the expression of genes that are largely unrelated to differentiation. SCOUP can omit the effect of such genes by reducing the weight of their influence automatically in pseudo-time optimization. In contrast, the positions of cells in reduced space will be affected and the pseudo-time will vary with the presence of such genes. Moreover, the dispersion of RNA-seq is higher than that of qPCR, which influences the analyses.

**Table 1:** PIS for each method applied to Shalek’s data

	LPS	PIC	PAM
SCOUP	0.03	0.12	0.12
SP	0.14	0.32	0.17
Monocle(2)	NA	0.38	NA
Monocle(3)	0.18	0.45	0.32
TSCAN	0.17	0.27	0.24

Each row represents the method, and each column represents the kind of stimulation for differentiation. NA means that Monocle did not work well

The PISs of PIC and PAM were higher than those of LPS. This will be because the numbers of PIC and PAM cells were lower than that of LPS. It is difficult to reconstruct differentiation trajectories from a small number of samples. In particular, it is important to obtain cells distributed evenly throughout the differentiation process in order to reconstruct trajectories with high accuracy.

In summary, SCOUP estimated pseudo-time with high accuracy, especially for RNA-seq data. Moreover, SCOUP successfully identified the initial state which was difficult to be detected with dimension reduction-based approaches. In addition, SCOUP is based on a probabilistic model, and hence can evaluate proper pseudo-time by using likelihood. Thus, SCOUP has advantages over dimension reduction-based methods in pseudo-time estimation.

### Validation of cell lineage estimate

In this section, we evaluate the accuracy of cell lineage estimation from single-cell expression data containing lineage bifurcation.

**Fig. 5:**
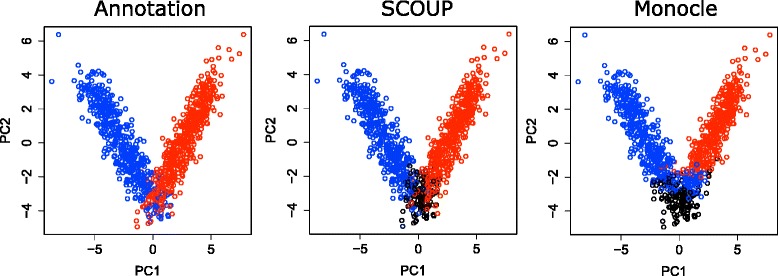
PCA of cells of Kouno’s data based on gene expression. The cell colors indicate the genuine lineage (*left*), lineage estimated with SCOUP (*middle*), and lineage estimated with Monocle (*right*). The color for SCOUP is defined by *γ*_*c*0_; black, 0.5; red, 0.0; and blue, 1.0. The color for Monocle is defined by estimated states: black, state 1 (pre-bifurcation); red, state 2; and blue, state 3. The color of each state is defined to be consistent among each plots

First, we validated SCOUP and Monocle with simulation data (Kouno’s data (2)). Table 2 shows the mean AUC values of each method for each condition. The AUC values for SCOUP were higher than those for Monocle in every condition. Figure 5 summarizes cells in the space of the first two PCs for expression data with *ε*=1.0. The color of each cell represents its genuine cell lineage (left), lineage estimated with SCOUP (middle), and lineage estimated with Monocle (right). Both methods estimated cell lineage with high accuracy for cells that were sufficiently separated in PCA space. This result suggests that estimating the lineage of a cell whose expression pattern has changed sufficiently after bifurcation is not difficult using these methods. However, Monocle was not able to estimate cell lineage correctly for cells whose expression pattern did not change sufficiently after bifurcation. In contrast, SCOUP successfully quantified the certainty of lineage of such cells and estimated their lineages with fairly high accuracy (Table 2). To understand cell fate decision mechanisms, it is important to analyze cells immediately after bifurcation. Therefore, SCOUP, which can quantify the certainty of estimated cell lineage and accurately estimate the lineage of cells that have just undergone bifurcation, will be useful for investigations of cell fate decision mechanisms.

**Table 2:** Mean AUC values for cell lineage estimates using each method for Kouno’s data (2)

	*ε*=0.0	*ε*=0.5	*ε*=1.0
SCOUP	0.99	0.99	0.99
Monocle	0.98	0.97	0.95

**Fig. 6:**
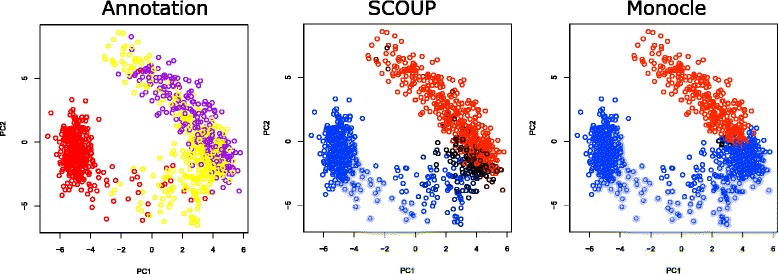
PCA of cells of Moignard’s data based on gene expression. The cell colors represent the genuine lineage (*left*), lineage estimated with SCOUP (*middle*), and lineage estimated with Monocle (*right*). The color for the genuine lineage is defined by the annotation of the cell; yellow, HF; red, 4SG; and purple, 4SFG^−^. The color for the SCOUP analysis is defined by *γ*_*c*0_; black, 0.5; red, 0.0; and blue, 1.0. The color for the Monocle analysis is defined by estimated states; black, state 1 (pre-bifurcation); red, state 2; and blue, state 3. We determined the color of each state to be consistent among each plot

Next, we investigated cell lineage estimation using Moignard’s data. The Moignard’s data includes the lineage bifurcation as follows; head fold (HF) cells differentiate into putative blood and endothelial populations, which are distinguished as either GFP^+^ cells (4SG) or Flk1^+^GFP^−^ cells (4SFG^−^). SCOUP was able to distinguish cells of 4SFG^−^ and 4SG almost completely correctly (AUC value = 1.00). This result did not change for Moignard’s data with all HF, 4SFG^−^ and 4SG cells (2,758 cells) (AUC value = 1.00). The AUC value of Monocle was 0.82. Figure 6 shows cells in the space of the first two PCs and the colors of cells indicate the genuine cell lineage (left), the lineage estimated using our method (middle), and the lineage using Monocle (right). The lineage estimation using SCOUP were highly consistent with cell annotations, while Monocle incorrectly regarded a non-negligible number of 4SFG^−^ cells as 4SG cells. This tendency of Monocle did not change when we changed the dimension number parameter (*max*_*components*). In contrast with simulation data, which were produced based on symmetric bifurcation, real data likely show complicated bifurcation patterns, and hence, a deterministic approach, such as MST in reduced space, might be inadequate to capture bifurcation.

The results described above show that SCOUP is superior to Monocle with respect to cell lineage estimation for both simulated and real data. SCOUP can capture subtle differences in cells immediately after bifurcation and will be a powerful method for investigations of cell fate decision mechanisms.

We also investigated cell lineage estimation with Gaussian mixture model (GMM) implemented in mclust package [24]. The AUC values for mclust were inferior to those of SCOUP, and mclust was not able to estimate cell lineage correctly for cells at an early stage of bifurcation (see Additional file 1 for AUC values and PCA plots of mclust). This is because mclust does not have time parameters in the model and will work well only for cells whose expression pattern has sufficiently changed after bifurcation. Moreover, GMM fitted to the position in which large number of cells exist for Moignard’s data. Therefore, GMM is inadequate to estimate the path of bifurcation in the condition that cells are unevenly distributed. Thus, it is important to take time parameters into account to estimate the path of differentiation and cell lineage.

### Clustering genes

**Fig. 7:**
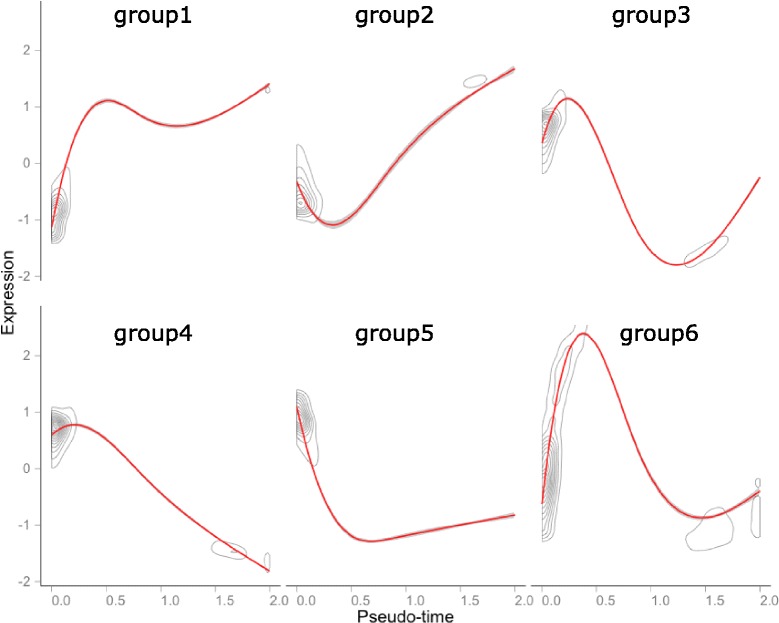
Overall trend in standardized expression patterns along pseudo-time for each group. This plot is drawn with the plot_clusters function in the Monocle package

We grouped genes for Shalek’s data based on expression patterns along pseudo-time estimated with SCOUP. Hereafter, we used the data for LPS stimulation because the number of LPS cells is largest in Shalek’s data. In this analysis, we investigated the top 5000 genes by the clustering method implemented in Monocle. Monocle regards the expression pattern as a function of pseudo-time and calculates a smooth response curve based on generalized additive models. Then, Monocle defines the distance between two genes as 1−*ρ*_*xy*_/2, where *ρ* is the Pearson correlation coefficient of standardized response curves, and groups genes with K-medoids clustering. In this analysis, we set the number of clusters as 6 and the overall trend in expression pattern for each cluster and the number of genes in each cluster are shown in Fig. 7 and Table 3.

**Table 3:** The number of top 5000 genes, top 1000 genes in each group. The total number are not equal to 5000 and 1000 because the response curves for a few genes could not be calculated

	Group					
	1	2	3	4	5	6
total	867	403	958	1354	778	599
top-1000 gene	260	81	177	291	76	111

We performed gene ontology (GO) enrichment analyses for genes in each group with DAVID [25, 26], and the top three GO terms (ordered by *p*-value) for each cluster are shown in Table 4. The cells of Shalek’s data are differentiated into dendritic cells, and immune response genes were upregulated (groups 1 and 2). Genes in groups 4 and 5 were downregulated and were enriched for the cell cycle GO term, consistent with previous research [27]. In this previous study, increased energy usage was also detected. In our analysis, genes related to energy usage were enriched in groups 3 and 6, which show a transient upregulation. Thus, we can classify gene function based on expression patterns along pseudo-time and the landscape of gene regulation can be characterized by investigating differences in these patterns. For example, although both groups 1 and 2 exhibited an upregulation, its timing was later for group 2 than group 1. The GO term related to “antigen” was enriched only in group 2, and this might reflect a different regulatory cascade during differentiation. We also calculated KEGG pathway enrichment for genes of group 1 and group 2, respectively. Group 2 did not include the term of KEGG pathway whose Benjamin-adjusted *p*-value was less than 10^−5^, wheres the term “Toll-like receptor signaling pathway” was the most significantly enriched in group 1 and Benjamin-adjusted *p*-value was 6.5×10^−7^. This data is the RNA-Seq of LPS stimulated bone-marrow derived dendritic cells and LPS is known to activate “Toll-like receptor signaling pathway” at first which cause the up-regulation of “antigen processing and presentation” a little late [28]. Our result is consistent with such mechanisms. Thus, investigations of expression patterns along pseudo-time can elucidate the regulatory machinery involved in differentiation.

**Table 4:** The top three GO terms for each group. The third column shows the negative logarithm of the Bonferroni-adjusted *p*-value

Group	GO term	−log10(*p*)
1	Immune response	22.9
	Defense response	11.4
	Response to wounding	7.0
2	Antigen processing and presentation	5.5
	Immune response	3.8
	Antigen processing and presentation of exogenous antigen	3.3
3	Generation of precursor metabolites and energy	5.1
	Protein localization	4.8
	Establishment of protein localization	3.2
4	Cell cycle	9.6
	Cell division	7.9
	Ribonucleoprotein complex biogenesis	7.7
5	Translation	6.7
	M phase of mitotic cell cycle	3.2
	Cell cycle	2.9
6	Generation of precursor metabolites and energy	11.5
	Protein transport	5.6
	Establishment of protein localization	5.5

### Correlation analysis

In this research, we propose a novel correlation analysis by using standardization based on SCOUP to detect covariance that cannot explained by the model that assumes the conditional independence among genes alone, and investigated the regulatory relationships among genes using correlations within raw expression data or standardized expression data. Hereafter, we refer to the correlations within raw data and standardized data as *C*_Raw_ and *C*_Std_, respectively. We first investigated whether the target genes of a transcription factor (TF) can be predicted under the assumption that the expression of a TF and its target genes are highly correlated. The list of TFs and their target genes was downloaded from the Integrated Transcription Factor Platform (ITFP) [29], a database containing 71 TFs and 648 pairs of TFs and target genes in the top 1000 genes. We calculated the *C*_Raw_ and *C*_Std_ values between 71 TFs and the remaining 929 genes and extracted from the top 1000 positively correlated pairs of TFs and genes according to each correlation method. The top 1000 *C*_Raw_ and *C*_Std_ values contained correlations of 24 and 27 annotated pairs, respectively (see Additional file 1 for the list of detected annotated pairs), and the probabilities of capturing these annotated pairs by random sampling are *p*<6.2×10^−5^ and *p*<2.8×10^−6^, respectively. This suggests that target genes of a specific TF can be predicted from a correlation analysis of single-cell expression data.

Only three annotated pairs were common between the 24 *C*_Raw_ correlation values and the 27 *C*_Std_ correlation values, which indicates that different regulatory relationships were detected when analyzing raw and standardized expression data. Analysis of standardized expression data revealed correlations that were not explained by the model that assumes the conditional independence among genes, whereas raw expression data analysis revealed correlations produced from similar expression patterns during differentiation. Thus, our novel correlation analysis method can deliver new insights that are not detected by conventional correlation methods.

Next, we aimed to detect a key regulator of each group by using the two correlation methods. We downloaded the candidates of key regulator TFs and their related genes from the Riken Transcription Factor Database (TFdb) [30] and FANTOM5 SSTAR [31] as well as TF data from ITFP. In this analysis, 117 genes of the annotated TFs and their related genes were contained in top 1000 gene and were considered as key regulator candidates. We calculated the *C*_Raw_ (and *C*_Std_) values between each candidate and genes in a group, and calculated the average *C*_Raw_ (*C*_Std_) value of the candidate for the group. We denote these values as $\overline {C}_{\text {Raw}}(i,j)$ and $\overline {C}_{\text {Std}}(i,j)$, where *i* is the index of a candidate and *j* is the index of a group. We assumed the key regulator of the group is highly correlated with genes in the group and investigated to detect the key regulators by extracting the candidates of high $\overline {C}_{\text {Raw}}(i,j)$ or $\overline {C}_{\text {Std}}(i,j)$. There were few differences between $\overline {C}_{\text {Raw}}(i,j)$ and $\overline {C}_{\text {Std}}(i,j)$ for groups 3 and 6 because our standardization was inadequate to deal with the transient patterns found in these groups. The difference between $\overline {C}_{\text {Raw}}(i,1)$ and $\overline {C}_{\text {Std}}(i,1)$ was largest among all groups, and therefore we focused on group 1 hereafter.

Table 5 shows the top three candidates according to $\overline {C}_{\text {Raw}}(i,1)$ and $\overline {C}_{\text {Std}}(i,1)$, respectively. The $\overline {C}_{\text {Raw}}(i,1)$ candidates are basically the genes which have large absolute expression differences between 1-h cells and 6-h cells. The large absolute expression difference can bring about high spurious correlation due to the similar expression trends during differentiation. Thus, *C*_Raw_ is likely to be influenced by spurious correlation and therefore is inadequate to detect the key regulator. As for $\overline {C}_{\text {Std}}(i,1)$, *Sqstm1* is the top rank. The absolute expression difference rank of *Sqstm1* is 313 of 1000 genes and the $\overline {C}_{\text {Raw}}(i,1)$ rank of *Sqstm1* is 29 of 117 candidates. Sqstm1, which is also called p62, has been suggested to be a key intracellular target of innate defense regulator peptides [32] and is therefore an important key factor for the immune response. Thus, our correlation method was able to detect a key factor that was difficult to detect by conventional correlation method and is a powerful tool for elucidating gene regulatory networks.

**Table 5:** The top three transcription factors and their related genes for group 1

Rank	Gene symbol	*C*_Raw_		Rank	Gene symbol	*C*_Std_
5	*Ifit1*	0.46		313	*Sqstm1*	0.076
6	*Ifi205*	0.44		45	*Ifih1*	0.071
17	*Ifi204*	0.43		5	*Ifit1*	0.071

The left and right tables correspond to $\overline {C}_{\text {Raw}}(i,1)$ and $\overline {C}_{\text {Std}}(i,1)$, respectively. The first column of each table contains the rank of the absolute difference of expression between 1-h cells and 6-h cells, and the second column lists the gene names. The third column contains the $\overline {C}_{\text {Raw}}(i,1)$ or ($\overline {C}_{\text {Std}}(i,1)$) of the candidate genes

**Fig. 8:**
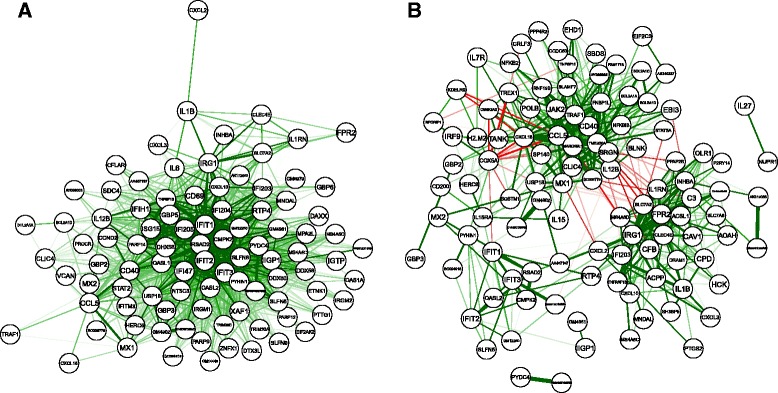
The correlation network based on *C*_Raw_ (**a**) and *C*_Std_ (**b**) for genes in group 1. There are a total of 93 and 107 genes in the *C*_Raw_ and *C*_Std_ network, respectively. The width of each edge represents the magnitude of an expression correlation between the two genes, and color represents the sign, green for a positive correlation and red for a negative correlation. To improve clarity, correlations with an absolute value lower than 0.55 (0.25) are not shown for *C*_Raw_ (*C*_Std_) network

Next, we investigated the correlation network for all genes in group 1 based on both the correlation methods. We omitted the genes with maximum of *C*_Raw_ (*C*_Std_) values lower than 0.6 (0.3) to improve visibility. Figure 8 show the correlation networks based on *C*_Raw_ (Fig. 8a) and *C*_Std_ (Fig. 8b). In the *C*_Raw_ network, the correlations of most of the gene pairs are positive because of spurious correlations over time, and most of the genes are therefore positively connected with each other. In contrast, the *C*_Std_ network mainly consists of two clusters, and there are a considerable number of negative correlations between the genes of different clusters. We assumed that each cluster is regulated by distinct regulatory mechanisms and investigated the differences of genes between two clusters. Hereafter, we focus on the chemokine genes (*CXCL2*, *CXCL3*, *CXCL10*, *CXCL16*, and *CCL5*), which are a family of small cytokines or proteins secreted by cells and are known to be involved in immune response [33]. In the *C*_Std_ network, *CXCL2*, *CXCL3*, and *CXCL10* belong to one cluster, while *CXCL16* and *CCL5* belong to another cluster. Although *CXCL16* belongs to the same CXC gene family, as *CXCL2*, *CXCL3*, and *CXCL10*, it has properties that distinguish it from other CXC chemokine genes. For example, *CXCL2*, *CXCL3*, and *CXCL10* are located in the proximal chromosomal region (5qE2, 5qE2, and 5qE3, respectively), while *CXCL16* is located on another chromosome (11qB4) [34]. Further, although *CCL5* belongs to a different gene family (the CC gene family), *CCL5* is located proximal to *CXCL16* (11qB5). The up-regulation of chemokine genes located in the proximal region has been suggested in breast cancer [35], and our correlation analysis also suggests that chemokine genes in located in the proximal region (*CXCL2*, *CXCL3*, and *CXCL10*) are regulated by different mechanisms than are *CXCL16* and *CCL5*. Thus, each clusters in the *C*_Std_ network is likely to be regulated by region-dependent mechanisms, and examining correlations among standardized gene expression profiles is a useful approach to elucidate regulatory networks that works by controlling for the effect of trends over time.

## Conclusions

The advancement of single-cell technologies will enable the elucidation of many biological processes, such as differentiation. The development of a novel computational method is necessary to fully analyze single-cell data. We developed a novel method, SCOUP, to analyze single-cell expression data for differentiation. Unlike previous methods, which use dimension reduction approaches and reconstruct differentiation trajectories in reduced space, SCOUP describes gene expression dynamics during differentiation directly, including pseudo-time and cell fate. We evaluated pseudo-time using SCOUP and previous methods based on the consistency between pseudo-time and experimental time and showed that the SCOUP results were superior to those of other methods for almost all conditions. We also compared the accuracy of cell lineage estimation using SCOUP and Monocle, and showed that SCOUP can estimate cell lineages with high accuracy, even for the cells at an early stage of bifurcation. SCOUP is based on a probabilistic model and can be extended to many applications. In this research, we developed a novel correlation analysis method based on SCOUP. It calculates the covariance that cannot be explained by a model, which assumes the conditional independence among genes, alone. We applied this method to scRNA-seq, and detected the candidate of key regulator of differentiation and the clusters in the correlation network which were not detected with conventional correlation analysis. In future work, we plan to extend our model to consider transient expression patterns complicated cell lineage pattern. In addition, we will develop a multivariate OU process to estimate gene regulatory networks more directly.

## Abbreviations

EM, expectation-maximization; GMM, gaussian mixture model; GO, gene ontology; LPS, lipopolysaccharide; MST, minimum spanning tree; OU, Ornstein–Uhlenbeck; PAM, synthetic mimic of a bacterial lipopeptides; PCA, principal component analysis; PIS, pseudo-time inconsistency score; PIC, viral-like double-stranded RNA

## Additional file

Additional file 1 Supplementary text. Full explanation and supplementary validation of SCOUP. (PDF 1040 kb)
