# Supplementary material for: SCOUP: a probabilistic model based on the Ornstein–Uhlenbeck process to analyze single-cell expression data during differentiation
Source: BMC Bioinformatics. 2016 Jun 8;17:232. doi: 10.1186/s12859-016-1109-3 (PMC4898467; doi:10.1186/s12859-016-1109-3)
Supplement: Additional file 1 — Supplementary text. Full explanation and supplementary validation of SCOUP. (PDF 1040 kb) [file 12859_2016_1109_MOESM1_ESM.pdf]

Supplementary Text for
SCOUP: a probabilistic model based on the Ornstein–Uhlenbeck process to analyze single-cell expression data during differentiation

Hirotaka Matsumoto, Hisanori Kiryu

March 15, 2016

1 Limit of a discrete time OU process

In this section, we consider limit of a discrete time OU process. Because both of genes and cells are supposed to be independent in SCOUP, we forget the index of gene and cell, and consider a general OU process in this section. We represent observed value as E and initial value as S, and $X = \{X_s | s = 0, ..., N\}$ is a path such that $X_N = E$ and $X_0 = S$. As mentioned in the main text, a OU process can be regarded as limit of a discrete time OU process:

$$P_{\mathrm{ou}}(E|S, \alpha, \sigma^2, t) = \lim_{N \to \infty} P_N(X_N | X_0, \alpha, \sigma^2, t)$$

$$P_N(X_N | X_0, \alpha, \sigma^2, t) = \int dX \prod_{s=1}^{N} P_{\mathrm{ou}}(X_s | X_{s-1}, \alpha, \sigma^2, t/N)$$

$$P(X|\alpha, \sigma^2, t) = \prod_{s=1}^{N} P_{\mathrm{ou}}(X_s | X_{s-1}, \alpha, \sigma^2, t/N) P(X_0),$$

where the interval of integration is the all paths which satisfies $X_0 = S$ and $X_N = E$.

Hereafter, we assume X_0 is given and re-define X as $X \in \{X_s | s = 1, ..., N\}$ for simplification. The calculation of the case that X_0 is unobserved is given in the after section. In this case, the complete likelihood is given by

$$P(X|S, \alpha, \sigma, t) = \prod_{s=1}^{N} P_{\mathrm{ou}}(X_s | X_{s-1}, \alpha, \sigma^2, t/N).$$

1.1 Transformation into the multivariate normal distribution

In this section, we transform the product of the transition probability $P_{\mathrm{ou}}(X_s | X_{s-1}, \alpha, \sigma^2, t/N)$ as the multivariate normal distribution. In the case of OU process, the transition probability is calculated with the normal distribution as follows:

$$P_{\mathrm{ou}}(X_s | X_{s-1}, \alpha, \sigma^2, t/N) = \sqrt{\frac{1}{2\pi V}} \exp\left(-\frac{1}{2V} (X_s - B X_{s-1} - (1 - B)\theta)^2 \right),$$

where

$$V = \frac{\sigma^2(1-\mathrm{e}^{-2\alpha t/N})}{2\alpha}, \quad B = \mathrm{e}^{-\alpha t/N}.$$

The complete likelihood $P(X|S,\alpha,\sigma^2,t)$ is equal to the following multivariate normal distribution:

$$\begin{aligned} P(X|S,\alpha,\sigma^2,t) &= \prod_{s=1}^{N} P_{\mathrm{ou}}(X_s|X_{s-1},\alpha,\sigma,t/N) \\ &= \frac{\sqrt{|\Lambda|}}{\left(\sqrt{2\pi}\right)^{N-1}} \exp\left(-\frac{1}{2}(X_{-N}-\mu)^{\mathrm{T}}\Lambda(X_{-N}-\mu)\right) \\ &= \mathcal{N}(X_{-N}|\mu,\Lambda^{-1}), \end{aligned}$$

where $X_{-N} \in \{X_s | s = 1, ..., N-1\}$ and Λ is $(N-1)\times(N-1)$ matrix and μ is $(N-1)$ dimension vector and satisfy following equations.

$$\Lambda_{i,j} = \begin{cases} \frac{1+B^2}{V} & (i=j) \\ -\frac{B}{V} & (j=i+1 \text{ or } j=i-1) \\ 0 & (\text{otherwise}) \end{cases}$$

$$\sum_{j=1}^{N-1} \Lambda_{i,j}\mu_j = \begin{cases} \frac{B}{V}X_0 + \frac{(1-B)^2}{V}\theta & (i=1) \\ \frac{(1-B)^2}{V}\theta & (1<i<N-1) \\ \frac{B}{V}X_N + \frac{(1-B)^2}{V}\theta & (i=N-1) \end{cases}$$

From above equation, μ_j can be calculated as follows:

$$\mu_j = \frac{B}{V}X_0\Lambda^{-1}_{1,j} + \frac{(1-B)^2}{V}\theta\sum_{i=1}^{N-1}\Lambda^{-1}_{i,j} + \frac{B}{V}X_N\Lambda^{-1}_{N-1,j}.$$

1.2 Derivation of mean vector and variance-covariance matrix

As mentioned in the next section, the expectation of X_s, X_sX_{s+1}, and X_s^2 are necessary to optimize parameters. These expectations can be calculated from the mean vector and variance-covariance matrix of the multivariate normal distribution. Because we consider a limit of a discrete time OU process, we cannot use numerical calculation and have to solve analytically. In this section, we derivate the mean vector and the variance-covariance matrix.

Firstly, we derivate the variance-covariance matrix. To simplify this, we define Λ' so that $\Lambda = -V^{-1}B\Lambda'$. We also define following variable:

$$\begin{aligned} -B^{-1} - B &= -(\mathrm{e}^{\alpha t/N} + \mathrm{e}^{-\alpha t/N}) = -2\cosh(\lambda) \\ \lambda &= \alpha t/N, \end{aligned}$$

and Λ' is represented as follows:

$$\Lambda'_{i,j} = \begin{cases} -2\cosh\lambda & (i=j) \\ 1 & (j=i+1 \text{ or } j=i-1) \\ 0 & (\text{otherwise}) \end{cases}$$

It is shown that the inversion of symmetric tridiagonal matrix can be calculated analytically [1]. By using this, we can derive the inversion of Λ' and Λ as follows:

$$[\Lambda']^{-1}_{i,j} = -\frac{\cosh(N-|j-i|)\lambda - \cosh(N-i-j)\lambda}{2\sinh\lambda\sinh N\lambda}$$
$$[\Lambda]^{-1}_{i,j} = \frac{V}{B}\frac{\cosh(N-|j-i|)\lambda - \cosh(N-i-j)\lambda}{2\sinh\lambda\sinh N\lambda}.$$

Next, we substitute Λ^{-1} and derive the mean μ_j.

$$\mu_j = V^{-1}BX_0\Lambda^{-1}_{1,j} + V^{-1}(1-B)^2\theta\sum_{i=1}^{N-1}\Lambda^{-1}_{i,j} + V^{-1}BX_N\Lambda^{-1}_{N-1,j}$$

Firstly, we solve the first member $V^{-1}BX_0\Lambda^{-1}_{1,j}$.

$$V^{-1}BX_0\Lambda^{-1}_{1,j} = \frac{\cosh(N-j+1)\lambda - \cosh(N-j-1)\lambda}{2\sinh\lambda\sinh N\lambda}X_0$$
$$= \frac{\sinh(N-j)\lambda}{\sinh N\lambda}X_0$$

Secondly, we solve third member $V^{-1}BX_N\Lambda^{-1}_{N-1,j}$.

$$V^{-1}BX_N\Lambda^{-1}_{1,N-1} = \frac{\cosh(j+1)\lambda - \cosh(-j+1)\lambda}{2\sinh\lambda\sinh N\lambda}X_N$$
$$= \frac{\sinh j\lambda}{\sinh N\lambda}X_N$$

Lastly, we solve $\sum_{i=1}^{N-1}\Lambda^{-1}_{i,j}$.

$$\sum_{i=1}^{N-1}\Lambda^{-1}_{i,j}$$
$$= \frac{V}{2B\sinh\lambda\sinh N\lambda}\sum_{i=1}^{N-1}(\cosh(N-|j-i|)\lambda - \cosh(N-i-j)\lambda)$$
$$= \frac{V}{2B\sinh\lambda\sinh N\lambda}\left(\sum_{i=1}^{j}(\cosh(N-j+i)\lambda - \cosh(N-i-j)\lambda) + \sum_{i=j+1}^{N-1}(\cosh(N+j-i)\lambda - \cosh(N-i-j)\lambda)\right)$$

Here, we use following equation to calculate above formula.

$$\sum_{i=1}^{j}(\cosh(N-j+i)\lambda - \cosh(N-i-j)\lambda)$$
$$= \frac{2}{(1-e^{\lambda})(1-e^{-\lambda})}(\sinh(N-j)\lambda\sinh j\lambda + \sinh(N-j)\lambda\sinh(j+1)\lambda + \sinh(N-j)\lambda\sinh\lambda)$$
$$\sum_{i=j+1}^{N-1}(\cosh(N+j-i)\lambda - \cosh(N-i-j)\lambda)$$
$$= \frac{2}{(1-e^{\lambda})(1-e^{-\lambda})}(\sinh j\lambda\sinh(N-j-1)\lambda - \sinh j\lambda\sinh(N-j)\lambda + \sinh j\lambda\sinh\lambda)$$

The sum of the above equations becomes

$$\frac{2}{(1-\mathrm{e}^{\lambda})(1-\mathrm{e}^{-\lambda})}(\sinh\lambda(\sinh(N-j)\lambda+\sinh j\lambda)+\sinh j\lambda\sinh(N-j-1)\lambda-\sinh(j+1)\lambda\sinh(N-j)\lambda).$$

By using following equation,

$$\sinh j\lambda\sinh(N-j-1)\lambda-\sinh(j+1)\lambda\sinh(N-j)\lambda=-\sinh\lambda\sinh N\lambda,$$

the sum can be described as follows:

$$\frac{2\sinh\lambda}{(1-\mathrm{e}^{\lambda})(1-\mathrm{e}^{-\lambda})}(\sinh(N-j)\lambda+\sinh j\lambda-\sinh N\lambda).$$

Therefore, second member $V^{-1}(1-B)^2\frac{V}{2B\sinh\lambda\sinh N\lambda}\theta\sum\Lambda$ becomes

$$\begin{aligned}&\frac{(1-B)^2}{2B\sinh N\lambda}\left(\frac{2\sinh\lambda}{(1-e^{\lambda})(1-e^{-\lambda})}(\sinh(N-j)\lambda+\sinh j\lambda-\sinh N\lambda)\right)\theta\\&=\frac{1}{\sinh N\lambda}\left(-\sinh(N-j)\lambda-\sinh j\lambda+\sinh N\lambda\right)\theta.\end{aligned}$$

Thus, the mean becomes

$$\begin{aligned}\mu_j&=\frac{\sinh(N-j)\lambda}{\sinh N\lambda}X_0+\frac{\sinh j\lambda}{\sinh N\lambda}X_N+\frac{\theta}{\sinh N\lambda}\left(-\sinh(N-j)\lambda-\sinh j\lambda+\sinh N\lambda\right)\\&=\frac{(X_0-\theta)\sinh(N-j)\lambda+(X_N-\theta)\sinh j\lambda}{\sinh N\lambda}+\theta.\end{aligned}$$

1.3 The complete log-likelihood

The complete log-likelihood of X given X_0 is given by

$$\begin{aligned}l(X)=&-\frac{N}{2}\ln\frac{V}{\pi}-\frac{V^{-1}(1+B^2)}{2}\sum_{s=1}^{N-1}X_s^2+V^{-1}B\sum_{s=0}^{N-1}X_sX_{s+1}+V^{-1}(1-B)^2\theta\sum_{s=1}^{N-1}X_s\\&-\frac{V^{-1}B^2}{2}X_0^2-V^{-1}B(1-B)\theta X_0-\frac{V^{-1}}{2}X_N^2+V^{-1}(1-B)\theta X_N-\frac{1}{2}\sum_{s=1}^{N}V^{-1}(1-B)^2\theta^2\\=&-\frac{N}{2}\ln\frac{\alpha}{\pi\sigma^2(1-\mathrm{e}^{-2\alpha t})}-\frac{N}{2t\sigma^2}\left(2\left(\sum_{s=1}^{N-1}X_s^2-\sum_{s=0}^{N-1}X_sX_{s+1}\right)+X_0^2+X_N^2\right)\\&+\frac{\alpha}{2\sigma^2}\left(X_0^2-X_N^2-2\theta X_0+2\theta X_N+\frac{\alpha t}{N}\left(-2\sum_{s=1}^{N-1}X_s^2+\sum_{s=0}^{N-1}X_sX_{s+1}+2\theta\sum_{s=1}^{N-1}X_s-N\theta^2\right)\right)+\mathcal{O}(1/N),\end{aligned}$$

and Q function is given by

$$
\begin{aligned}
\mathcal{Q} =& -\frac{N}{2}\ln\frac{V}{\pi} - \frac{V^{-1}(1+B^2)}{2}\sum_{s=1}^{N-1} < X_s^2 > + V^{-1}B\sum_{s=0}^{N-1} < X_sX_{s+1} > + V^{-1}(1-B)^2\theta\sum_{s=1}^{N-1} < X_s > \\
& - \frac{V^{-1}B^2}{2}X_0^2 - V^{-1}B(1-B)\theta X_0 - \frac{V^{-1}}{2}X_N^2 + V^{-1}(1-B)\theta X_N - \frac{1}{2}NV^{-1}(1-B)^2\theta^2 \\
=& -\frac{N}{2}\ln\frac{\alpha^*}{\pi\sigma^{*2}(1-\mathrm{e}^{-2\alpha^* t})} - \frac{N}{2t^*\sigma^{*2}}(2F_{ss} - 2F_{ss+1} + X_0^2 + X_N^2) \\
& + \frac{\alpha^*}{2\sigma^{*2}}\left(X_0^2 - X_N^2 - 2\theta^* X_0 + 2\theta^* X_N + \frac{\alpha^* t^*}{N}\left(-2F_{ss} + F_{ss+1} + 2F_s - N\theta^{*2}\right)\right) + \mathcal{O}(1/N),
\end{aligned}
$$

where F_{ss}, F_{ss+1}, F_s is $\sum_{s=1}^{N-1} < X_s^2 >, \sum_{s=0}^{N-1} < X_sX_{s+1} >, \sum_{s=1}^{N-1} < X_s >$, respectively.

1.4 Derivation of the sufficient statistic

The likelihood function depends on X only through X_s^2, X_sX_{s+1}, and X_s, these are the sufficient statistic for the model. In this section, we derive the these statistic analytically.

1.4.1 Derivation of F_{ss}

Firstly, we solve the expectation of X_s^2.

$$
\sum_{s=1}^{N-1} < X_s^2 > = \sum_{s=1}^{N-1}\Lambda_{ss}^{-1} + \sum_{s=1}^{N-1}\mu_s^2
$$

The first member is

$$
\begin{aligned}
\sum_{s=1}^{N-1}\Lambda_{ss}^{-1} &= \frac{V}{2B\sinh\lambda\sinh N\lambda}\sum(\cosh N\lambda - \cosh(N-2s)\lambda) \\
&\simeq \frac{\sigma^2}{2\alpha\sinh N\lambda}\left(N\cosh N\lambda - \frac{1}{\lambda}\sinh N\lambda - \lambda\sinh N\lambda\right)
\end{aligned}
$$

The second member is

$$
\begin{aligned}
\sum_{s=1}^{N-1}\mu_s^2 =& \sum\left(\frac{(X_0-\theta)\sinh(N-s)\lambda + (X_N-\theta)\sinh s\lambda}{\sinh N\lambda} + \theta\right)^2 \\
=& \frac{(X_0-\theta)^2 + (X_N-\theta)^2}{\sinh^2 N\lambda}\sum\sinh^2 s\lambda + \frac{2(X_0-\theta)(X_N-\theta)}{\sinh^2 N\lambda}\sum\sinh s\sinh(N-s)\lambda \\
& + \frac{2\theta(X_0+X_N-2\theta)}{\sinh N\lambda}\sum\sinh s\lambda + (N-1)\theta^2
\end{aligned}
$$

1.4.2 Derivation of F_{ss+1}

Secondly, we calculate the following equation:

$$
\sum_{s=0}^{N-1} < X_sX_{s+1} > = \sum_{s=1}^{N-2}\Lambda_{ss+1}^{-1} + \sum_{s=1}^{N-2}\mu_s\mu_{s+1} + \mu_1X_0 + \mu_{N-1}X_N.
$$

The first member is

$$\sum_{s=1}^{N-2} \Lambda^{-1}_{ss+1} = \frac{V}{2B\sinh\lambda\sinh N\lambda}\sum(\cosh(N-1)\lambda - \cosh(N-2s-1)\lambda)$$
$$\simeq \frac{\sigma^2}{2\alpha\sinh N\lambda}\left(N\cosh N\lambda - \frac{1}{\lambda}\sinh N\lambda - N\lambda\sinh N\lambda - \frac{1}{2}\lambda\sinh N\lambda + \frac{N\lambda^2}{2}\cosh N\lambda\right).$$

The remainder is

$$\sum_{s=1}^{N-2} \mu_s\mu_{s+1} + \mu_1 X_0 + \mu_{N-1}X_N$$
$$= \sum_{s=0}^{N-1}\left(\frac{(X_0-\theta)\sinh(N-s)\lambda + (X_N-\theta)\sinh s\lambda}{\sinh N\lambda} + \theta\right)\left(\frac{(X_0-\theta)\sinh(N-s-1)\lambda + (X_N-\theta)\sinh(s+1)\lambda}{\sinh N\lambda} + \theta\right)$$
$$= \frac{(X_0-\theta)^2 + (X_N-\theta)^2}{\sinh^2 N\lambda}\left(\cosh\lambda\sum_{s=1}^{N-1}\sinh^2 s\lambda + \frac{\sinh\lambda}{2}\sum_{s=1}^{N-1}\sinh 2\lambda\right)$$
$$+ \frac{(X_0-\theta)(X_N-\theta)}{\sinh^2 N\lambda}\left(2\cosh\lambda\sum_{s=1}^{N-1}\sinh s\lambda\sinh(N-s)\lambda + \sinh N\lambda\sinh\lambda\right)$$
$$+ \frac{\theta(X_0+X_N-2\theta)}{\sinh N\lambda}\left(2\sum_{s=1}^{N-1}\sinh s\lambda + \sinh N\lambda\right) + N\theta^2.$$

1.4.3 Derivation of F_s

Lastly, we calculate F_s.

$$\sum_{s=1}^{N-1} < X_s > = \sum\left(\frac{(X_0-\theta)\sinh(N-s)\lambda + (X_N-\theta)\sinh s\lambda}{\sinh N\lambda} + \theta\right)$$
$$= \frac{X_0 + X_N - 2\theta}{\sinh N\lambda}\sum_{s=1}^{N-1}\sinh s\lambda + (N-1)\theta$$

1.5 Derivation of Q function

As mentioned in the above section, Q function is

$$\mathcal{Q} = -\frac{N}{2}\ln\frac{\alpha^*}{\pi\sigma^{*2}(1-e^{-2\alpha^* t})} - \frac{N}{2t^*\sigma^{*2}}(2F_{ss} - 2F_{ss+1} + X_0^2 + X_N^2)$$
$$+ \frac{\alpha^*}{2\sigma^{*2}}\left(X_0^2 - X_N^2 - 2\theta^* X_0 + 2\theta^* X_N + \frac{\alpha^* t^*}{N}\left(-2F_{ss} + F_{ss+1} + 2F_s - N\theta^{*2}\right)\right) + \mathcal{O}(1/N).$$

In the following section, we calculate the Q function.

1.5.1 Derivation of $2F_{ss} - 2F_{ss+1} + X_0^2 + X_N^2$

Firstly, we calculate the member which is related to $\sum \Lambda_{ss} - \sum \Lambda_{ss+1}$.

$$\begin{aligned}2\sum \Lambda_{ss} - 2\sum \Lambda_{ss+1} &\simeq \frac{\sigma^2}{2\alpha \sinh N\lambda}(2N\lambda \sinh N\lambda - \lambda \sinh N\lambda - N\lambda^2 \cosh N\lambda) \\ &= t\sigma^2 - \lambda \left(\frac{\sigma^2}{2\alpha} + \frac{t\sigma^2 \cosh N\lambda}{2 \sinh N\lambda} \right)\end{aligned}$$

Secondary, we calculate the member which is related to $(X_0 - \theta)^2 + (X_N - \theta)^2$.

$$\begin{aligned}&\frac{1}{\sinh^2 N\lambda}(2\sum \sinh^2 s\lambda - 2\cosh \lambda \sum \sinh^2 s\lambda - \sinh \lambda \sum \sinh 2\lambda) \\ &\simeq -1 + \lambda \left(\frac{\cosh N\lambda}{2 \sinh N\lambda} + \frac{N\lambda}{2 \sinh^2 N\lambda} \right)\end{aligned}$$

Thirdly, we calculate the member which is related to $(X_0 - \theta)(X_N - \theta)$.

$$\begin{aligned}&\frac{1}{\sinh^2 N\lambda} \left(4\sum \sinh s\lambda \sinh(N-s)\lambda - 4\cosh \lambda \sum \sinh s\lambda \sinh(N-s)\lambda + 2\sinh N\lambda \sinh \lambda \right) \\ &\simeq -\frac{\lambda}{\sinh^2 N\lambda} (\sinh N\lambda + N\lambda \cosh N\lambda)\end{aligned}$$

Fourthly, we calculate the member which is related to $\theta(X_0 + X_N - 2\theta)$.

$$\frac{1}{\sinh N\lambda} \left(4\sum \sinh s\lambda - 4\sum \sinh s\lambda - 2\sinh N\lambda \right) = -2$$

Lastly, we calculate the member which is related to θ^2.

$$2(N-1) - 2N = -2$$

With above calculation results, $2F_{ii} - 2F_{ii+1} + X_0^2 + X_N^2$ can be derived as follows:

$$\begin{aligned}&t\sigma^2 - \lambda \left(\frac{\sigma^2}{2\alpha} + \frac{N\lambda\sigma^2 \cosh N\lambda}{2\alpha \sinh N\lambda} \right) - (X_0 - \theta)^2 - (X_N - \theta)^2 - 2\theta(X_0 + X_N - 2\theta) - 2\theta^2 + X_0^2 + X_N^2 \\ &+ \lambda \left(\frac{\cosh N\lambda}{2 \sinh N\lambda} + \frac{N\lambda}{2 \sinh^2 N\lambda} \right) ((X_0 - \theta)^2 + (X_N - \theta)^2) \\ &- \frac{\lambda}{\sinh^2 N\lambda} (\sinh N\lambda + N\lambda \cosh N\lambda) (X_0 - \theta)(X_N - \theta) \\ =&t\sigma^2 - \lambda \left(\frac{\sigma^2}{2\alpha} - \frac{N\lambda\sigma^2 \cosh N\lambda}{2\alpha \sinh N\lambda} \right) \\ &+ \lambda \left(\frac{\cosh N\lambda}{2 \sinh N\lambda} + \frac{N\lambda}{2 \sinh^2 N\lambda} \right) ((X_0 - \theta)^2 + (X_N - \theta)^2) \\ &- \frac{\lambda}{\sinh^2 N\lambda} (\sinh N\lambda + N\lambda \cosh N\lambda) (X_0 - \theta)(X_N - \theta).\end{aligned}$$

1.5.2 Derivation of $X_0^2 - X_N^2 - 2\theta^* X_0 + 2\theta^* X_N + \lambda^*(-2F_{ss} + F_{ss+1} + 2\theta^* F_s - N\theta^{*2})$

In this section, we calculate the coefficient of $\frac{\alpha^*}{2\sigma^{*2}}$ which is $X_0^2 - X_N^2 - 2\theta^* X_0 + 2\theta^* X_N + \lambda^*(-2F_{ss} + F_{ss+1} + 2\theta^* F_s - N\theta^{*2})$. In this calculation, we disregard the $1/N$ order terms.

At first, we calculate the member related to F_{ss} and F_{ss+1}. The member related to Λ is calculated as follows:

$$\begin{aligned}
-2\lambda^* \sum \Lambda_{ss} + \lambda^* \sum \Lambda_{ss+1} &\simeq -\frac{\lambda^* \sigma^2}{2\alpha \sinh N\lambda} \left(N \cosh N\lambda - \frac{1}{\lambda} \sinh N\lambda \right) \\
&= \frac{\lambda^*}{\lambda} \left(\frac{\sigma^2}{2\alpha} - \frac{t\sigma^2 \cosh N\lambda}{2 \sinh N\lambda} \right).
\end{aligned}$$

The member related to $(X_0 - \theta)^2 + (X_N - \theta)^2$ is

$$\begin{aligned}
&- \frac{\lambda^*}{\sinh^2 N\lambda} \left(2 \sum \sinh^2 s\lambda - \cosh \lambda \sum \sinh^2 s\lambda - \frac{\sinh \lambda}{2} \sum \sinh 2\lambda \right) \\
&\simeq \frac{\lambda^*}{\lambda} \left(-\frac{\cosh N\lambda}{2 \sinh N\lambda} + \frac{N\lambda}{2 \sinh^2 N\lambda} \right).
\end{aligned}$$

The member related to $(X_0 - \theta)(X_N - \theta)$ is

$$\begin{aligned}
&- \frac{\lambda^*}{\sinh^2 N\lambda} \left(4 \sum \sinh i\lambda \sinh(N - s)\lambda - 2 \cosh \lambda \sum \sinh s\lambda \sinh(N - s)\lambda - \sinh N\lambda \sinh \lambda \right) \\
&\simeq \frac{\lambda^*}{\lambda} \frac{1}{\lambda \sinh^2 N\lambda} \left(\sinh N\lambda - N\lambda \cosh N\lambda \right).
\end{aligned}$$

The member related to $\theta(X_0 + X_N - 2\theta)$ is

$$-\frac{\lambda^*}{\sinh N\lambda} \left(4 \sum \sinh s\lambda - 2 \sum \sinh s\lambda - \sinh N\lambda \right) \simeq -2 \frac{\lambda^*}{\lambda} \frac{\cosh N\lambda - 1}{\lambda \sinh N\lambda}.$$

The member related to $\theta^*(X_0 + X_N - 2\theta)$ is

$$\lambda^* \frac{2}{\sinh N\lambda} \sum \sinh s\lambda \simeq 2 \frac{\lambda^*}{\lambda} \frac{\cosh N\lambda - 1}{\sinh N\lambda}.$$

And the member related to $\theta^2, \theta^*\theta, \theta^{*2}$ is

$$\lambda^* \left((-2(N-1) + N)\theta^{*2} + 2(N-1)\theta\theta^* - N\theta^2 \right) \simeq -N\lambda^*(\theta^* - \theta)^2.$$

Therefore, $X_0^2 - X_N^2 - 2\theta^* X_0 + 2\theta^* X_N + \lambda^*(-2F_{ss} + F_{ss+1} + 2\theta^* F_s - N\theta^{*2})$ becomes as follows:

$$\begin{aligned}
&(X_0 - \theta^*)^2 - (X_N - \theta^*)^2 + \frac{\lambda^*}{\lambda} \left(-\frac{\cosh N\lambda}{2 \sinh N\lambda} + \frac{N\lambda}{2 \sinh^2 N\lambda} \right) ((X_0 - \theta)^2 + (X_N - \theta)^2) \\
&+ \frac{\lambda^*}{\lambda} \frac{1}{\sinh^2 N\lambda} \left(\sinh N\lambda - N\lambda \cosh N\lambda \right) (X_0 - \theta)(X_N - \theta) + 2 \frac{\lambda^*}{\lambda} \frac{\cosh N\lambda - 1}{\sinh N\lambda} (\theta^* - \theta)(X_0 + X_N - 2\theta) \\
&- N\lambda^*(\theta^* - \theta)^2 + \frac{\lambda^*}{\lambda} \left(\frac{\sigma^2}{2\alpha} - \frac{t\sigma^2 \cosh N\lambda}{2\alpha \sinh N\lambda} \right),
\end{aligned}$$

1.6 Q function

The standardization term is approximated as follows:

$$\frac{2\alpha^*}{1-\mathrm{e}^{-2\alpha^* t/N}} \simeq \frac{N}{t}\frac{1}{1-\frac{\alpha^* t}{N}} \simeq \frac{N}{t} + \alpha^*.$$

By using the calculation results so far, the Q function is described as follows:

$$\begin{aligned}
\mathcal{Q} =& \frac{N}{2}\ln\left(\frac{N}{t^*}+\alpha^*\right) - \frac{N}{2}\ln\sigma^{*2} - \frac{Nt\sigma^2}{2t^*\sigma^{*2}} + \frac{\alpha^*}{2\sigma^{*2}}\left(\frac{\alpha t}{\alpha^* t^*}+\frac{\alpha^* t^*}{\alpha t}\right)\frac{\sigma^2}{2\alpha} + \frac{\alpha^*}{2\sigma^{*2}}\left(\frac{\alpha t}{\alpha^* t^*}-\frac{\alpha^* t^*}{\alpha t}\right)\frac{t\sigma^2\cosh N\lambda}{2\sinh N\lambda} \\
&+ \frac{\alpha^*}{2\sigma^{*2}}\left(\frac{\alpha t}{\alpha^* t^*}+\frac{\alpha^* t^*}{\alpha t}\right)\left(-\frac{\cosh N\lambda}{2\sinh N\lambda}((X_0-\theta)^2+(X_N-\theta)^2)+\frac{1}{\sinh N\lambda}(X_0-\theta)(X_N-\theta)\right) \\
&+ \frac{\alpha^*}{2\sigma^{*2}}\left(\frac{\alpha t}{\alpha^* t^*}-\frac{\alpha^* t^*}{\alpha t}\right)\left(-\frac{N\lambda}{2\sinh^2 N\lambda}((X_0-\theta)^2+(X_N-\theta)^2)+\frac{N\lambda\cosh N\lambda}{\sinh^2 N\lambda}(X_0-\theta)(X_N-\theta)\right) \\
&+ \frac{\alpha^*}{2\sigma^{*2}}\frac{\alpha^* t^*}{\alpha t}(\theta^*-\theta)\left(2\frac{\cosh N\lambda-1}{\sinh N\lambda}(X_0+X_N-2\theta)\right) - \frac{\alpha^{*2}t^*}{2\sigma^{*2}}(\theta^*-\theta)^2 \\
&+ \frac{\alpha^*}{2\sigma^{*2}}((X_0-\theta^*)^2-(X_N-\theta^*)^2)+\mathcal{O}(1/N).
\end{aligned}$$

1.7 Parameter optimization

We optimize parameters by solving $d\mathcal{Q}/d\theta^*=0, d\mathcal{Q}/d\alpha^*=0, d\mathcal{Q}/d\sigma^{*2}=0$, and $d\mathcal{Q}/dt^*=0$. In this research, we optimize all parameters independently.

1.7.1 Optimization of θ

We solve $d\mathcal{Q}/d\theta^*=0$.

$$d\mathcal{Q}/d\theta^* = \frac{\alpha}{2\sigma^2}\left(2\frac{\cosh\alpha t-1}{\sinh\alpha t}(X_0+X_N-2\theta)-2\alpha(\theta^*-\theta)-2(X_0-X_N)\right)$$

So, θ^* which satisfies $d\mathcal{Q}/d\theta^*=0$ is

$$\begin{aligned}
\theta^* &= \theta+\frac{1}{\alpha t}\left(\frac{\cosh\alpha t-1}{\sinh\alpha t}(X_0+X_N-2\theta)-(X_0-X_N)\right) \\
&= \theta+\frac{2}{\alpha t(1+\mathrm{e}^{-2\alpha t})}\left(X_N-\mathrm{e}^{-\alpha t}X_0-(1-\mathrm{e}^{-\alpha t})\theta\right).
\end{aligned}$$

1.7.2 Optimization of α

α^* which satisfies $d\mathcal{Q}/d\alpha^*=0$ is

$$\alpha^* = \frac{-\sigma^2 t+(X_N-\theta)^2-(X_0-\theta)^2}{Z^\alpha},$$

where

$$
\begin{aligned}
Z^\alpha =& \frac{\sigma^2}{\alpha}\left(\frac{1}{\alpha} - \frac{t\cosh\alpha t}{\sinh\alpha t}\right) + \frac{2}{\alpha}\left(\frac{\alpha t}{2\sinh^2\alpha t} - \frac{\cosh\alpha t}{2\sinh\alpha t}\right)((X_0-\theta)^2 + (X_N-\theta)^2) \\
&+ \frac{2}{\alpha}\left(\frac{1}{\sinh\alpha t} - \frac{\alpha t\cosh\alpha t}{\sinh^2\alpha t}\right)(X_0-\theta)(X_N-\theta).
\end{aligned}
$$

1.7.3 Optimization of σ^2

We solve $d\mathcal{Q}/d\sigma^{*2} = 0$.

$$
\sigma^{*2} = \sigma^2 - \frac{1}{N}\left(\sigma^2 + 2\alpha\left(-\frac{\cosh\alpha t}{2\sinh\alpha t}((X_0-\theta)^2 + (X_N-\theta)^2) + \frac{1}{\sinh\alpha t}(X_0-\theta)(X_N-\theta)\right) + \alpha((X_0-\theta)^2 - (X_N-\theta)^2)\right)
$$

The highest order term results in $\sigma^{*2} = \sigma^2$ which means σ^2 will not change. Therefore, we optimize in regards to second highest term.

$$
\sigma^2 = \frac{2\alpha}{1-\mathrm{e}^{-2\alpha t}}\left(X_N - \mathrm{e}^{-\alpha t}X_0 - (1-\mathrm{e}^{-\alpha t})\theta\right)^2
$$

When we regard above σ^2 as σ^{*2}, $d\mathcal{Q}/d\sigma^{*2}$ will be zero up to second highest order if σ^2 is converged sufficiently.

1.7.4 Optimization of t

We solve $d\mathcal{Q}/dt^* = 0$. Because t^* is related to α and σ, we consider $\alpha^{'*} = t^*\alpha, \sigma^{'*2} = t^*\sigma^2$.

$$
\begin{aligned}
\frac{d\mathcal{Q}}{d\alpha^{'*}} &= \frac{1}{2} + \frac{1}{2\alpha t} - \frac{1}{2}\frac{\sinh\alpha t}{\cosh\alpha t} + \frac{1}{t\sigma_g^2}(D^{(1)} - D^{(2)}) + \frac{1}{2t^*\sigma^2}((X_0-\theta)^2 - (X_N-\theta)^2) \\
\frac{d\alpha^{'*}}{dt^*}\frac{d\mathcal{Q}}{d\alpha^{'*}} &= \frac{\alpha}{2} + \frac{1}{2t} - \frac{\alpha}{2}\frac{\sinh\alpha t}{\cosh\alpha t} + \frac{\alpha}{t\sigma^2}(D^{(1)} - D^{(2)}) + \frac{\alpha}{2t^*\sigma^2}((X_0-\theta)^2 - (X_N-\theta)^2),
\end{aligned}
$$

and

$$
\begin{aligned}
\frac{d\sigma^{'*2}}{dt^*}\frac{d\mathcal{Q}}{d\sigma^{'*2}} =& \frac{N}{2}\frac{1}{t^{*2}}(t-t^*) - \frac{1}{4t^{*2}}\left(t + \alpha t^2\frac{\sinh\alpha t}{\cosh\alpha t} + \frac{2\alpha t}{\sigma^2}(D^{(1)} + D^{(2)})\right) - \frac{\alpha}{2t^*\sigma^2}((X_0-\theta)^2 - (X_N-\theta)^2) \\
&- \frac{1}{4t^2}\left(t - \alpha t^2\frac{\sinh\alpha t}{\cosh\alpha t} + \frac{2\alpha t}{\sigma^2}(D^{(1)} - D^{(2)})\right),
\end{aligned}
$$

where

$$
\begin{aligned}
D^{(1)} &= -\frac{\cosh N\lambda}{2\sinh N\lambda}((X_0-\theta)^2 + (X_N-\theta)^2) + \frac{1}{\sinh N\lambda}(X_0-\theta)(X_N-\theta) \\
D^{(2)} &= -\frac{N\lambda}{2\sinh^2 N\lambda}((X_0-\theta)^2 + (X_N-\theta)^2) + \frac{N\lambda\cosh N\lambda}{\sinh^2 N\lambda}(X_0-\theta)(X_N-\theta)
\end{aligned}
$$

So, $d\mathcal{Q}/dt^*$ is described as follows:

$$
\begin{aligned}
\frac{d\mathcal{Q}}{dt^*} =& \frac{d\alpha'^*}{dt^*}\frac{dQ}{d\alpha'^*} + \frac{d\sigma'^{*2}}{dt^*}\frac{dQ}{d\sigma'^{*2}} \\
=& \frac{N}{2}\frac{1}{t^{*2}}(t - t^*) \\
& - \frac{1}{4t^{*2}}\left(t + \alpha t^2 \frac{\sinh\alpha t}{\cosh\alpha t} + \frac{2\alpha t}{\sigma^2}(D^{(1)} + D^{(2)})\right) - \frac{1}{4t^2}\left(-t + \alpha t^2\left(-2 + \frac{\sinh\alpha t}{\cosh\alpha t}\right) + \frac{2\alpha t}{\sigma^2}(-D^{(1)} + D^{(2)})\right).
\end{aligned}
$$

The highest order term results in $t^* = t$ and we consider second highest term. In the case that $d\mathcal{Q}/dt^* = 0$ for second highest order, the following equation consists.

$$
t + \alpha t^2 \frac{\sinh\alpha t}{\cosh\alpha t} + \frac{2\alpha t}{\sigma^2}(D^{(1)} + D^{(2)}) = t - \alpha t^2\left(-2 + \frac{\sinh\alpha t}{\cosh\alpha t}\right) + \frac{2\alpha t}{\sigma^2}(D^{(1)} - D^{(2)})
$$

Above equation becomes as follows:

$$
\alpha t\left(1 - \frac{\cosh\alpha t}{\sinh\alpha t}\right) - 2\frac{\alpha}{\sigma^2}E = 0.
$$

Unfortunately, t cannot be solved analytically and we use Newton's method.

$$
\begin{aligned}
f(t) =& \alpha t\left(1 - \frac{\cosh\alpha t}{\sinh\alpha t}\right) - 2\frac{\alpha}{\sigma^2}D^{(2)} \\
\frac{df(t)}{dt} =& \alpha\left(1 - \frac{\cosh\alpha t}{\sinh\alpha t}\right) + \frac{\alpha^2 t}{\sinh^2\alpha t} - 2\frac{\alpha^2}{\sigma^2}\frac{1}{\sinh^3\alpha t}\Big(- \frac{1}{2}(\sinh\alpha t - 2\alpha t\cosh\alpha t)((X_0 - \theta)^2 + (X_N - \theta)^2) \\
& + (\cosh\alpha t\sinh\alpha t - \alpha t(1 + \cosh^2\alpha t))(X_0 - \theta)(X_N - \theta)\Big) \\
t_{n+1} =& t_n - \frac{f(t_n)}{f'(t_n)}
\end{aligned}
$$

We optimize t iteratively by using above equation.

2 Mixture OU process for multi-lineage differentiation

We denote the number of cell, gene, and lineage by C, G, and K, respectively. We also denote the index of cell, gene, and lineage by c, g, and k, respectively. We assume each lineage has different attractor θ_{gk} and the likelihood is given by

$$
\begin{aligned}
P(E|S,\Theta,T) &= \prod_{c=1}^{C}\prod_{g=1}^{G} P(E_{gc}|S_{gc},\theta_g,t_c) \\
&= \prod_{c=1}^{C}\left(\sum_{k=1}^{K}\pi_k\prod_{g=1}^{G} P(E_{gc}|S_{gc},\theta_{gk},t_c)\right) \\
&= \prod_{c=1}^{C}\left(\sum_{k=1}^{K}\pi_k\prod_{g=1}^{G}\sum_{X_{gc}\in(E_{gc},S_{gc})}\prod_{s=1}^{N} P(X_{gcs}|X_{gcs-1},\theta_{gk},t_c/N)\right),
\end{aligned}
$$

where π_k is the probability of lineage k.

With the latent value Z_c which is 1 of K representation and indicates the cell fate, $P(X, Z|E, S, \Theta, T)$ and $P(Z|E, S, \Theta, T)$ are given by

$$P(X, Z|E, S, \Theta, T) \propto \prod_{c=1}^{C} \prod_{k=1}^{K} \left(\pi_k^{Z_{ck}} \prod_{g=1}^{G} \prod_{s=1}^{N} P(X_{gcs}|X_{gcs-1}, \theta_{gk}, t_c/N)^{Z_{ck}} \right)$$

$$P(Z|E, S, \Theta, T) \propto \prod_{c=1}^{C} \prod_{k=1}^{K} \left(\pi_k^{Z_{ck}} \prod_{g=1}^{G} P(E_{gc}|S_{gc}, \theta_{gk}, t_c)^{Z_{ck}} \right).$$

So, γ_{ck}, which is the expectation of posterior probability of Z_{ck} is represented as follows:

$$\gamma_{ck} = \mathrm{E}[Z_{ck}] = \frac{\pi_k \prod_{g=1}^{G} P(E_{gc}|S_{gc}, \theta_{gk}, t_c)}{\sum_{k'} \pi'_k \prod_{g=1}^{G} P(E_{gc}|S_{gc}, \theta_{gk'}, t_c)}.$$

To avoid overfitting, we added pseudo-count and re-defined γ_{ck} as follows:

$$\gamma_{ck} := \frac{\gamma_{ck} + 0.01}{\sum_{k'}(\gamma_{ck'} + 0.01)} = \frac{\gamma_{ck} + 0.01}{1 + 0.01 \times K}.$$

Hereafter, we denote $\ln P(X_{cg}|S_{cg}, Z_{ck} = 1)$ by l_{gck}, and l_{gck} is described as follows:

$$\begin{aligned} l_{gck} =& \ln \left(\prod_{i=1}^{N} P(X_{gcs}|X_{gcs-1}, \theta_{gk}, t_c/N) \right) \\ =& -\frac{N}{2} \ln \frac{V_g}{\pi} - \frac{V_g^{-1}(1 + B_g^2)}{2} \sum_{s=1}^{N-1} X_{gcs}^2 + V_g^{-1} B_g \sum_{s=1}^{N-2} X_{gcs} X_{gcs+1} + V_g^{-1}(1 - B_g)^2 \theta_{gk} \sum_{s=1}^{N-1} X_{gcs} \\ &+ V_g^{-1} B_g X_{gc0} X_{gc1} + V_g^{-1} B_g X_{gcN} X_{gcN-1} - \frac{V_g^{-1} B_g^2}{2} X_{gc0}^2 - V_g^{-1} B_g (1 - B_g) \theta_{gk} X_{gc0} \\ &- \frac{V_g^{-1}}{2} X_{gcN}^2 + V_g^{-1}(1 - B_g) \theta_{gk} X_{gcN} - \frac{1}{2} \sum_{i=1}^{N} V_g^{-1} (1 - B_g)^2 \theta_{gk}^2. \end{aligned}$$

And the Q function of l_{gck} ($\mathcal{Q}_{gck}$) is

$$\begin{aligned} \mathcal{Q}_{gck} =& \frac{N}{2} \ln \left(\frac{N}{t_c^*} + \alpha_g^* \right) - \frac{N}{2} \ln \sigma_g^{*2} - \frac{N t \sigma_g^2}{2 t_c^* \sigma_g^{*2}} \\ &+ \frac{\alpha_g^*}{2\sigma_g^{*2}} \left(\frac{\alpha_g t_c}{\alpha_g^* t_c^*} + \frac{\alpha_g^* t_c^*}{\alpha_g t_c} \right) \frac{\sigma_g^2}{2\alpha_g} + \frac{\alpha_g^*}{2\sigma_g^{*2}} \left(\frac{\alpha_g t_c}{\alpha_g^* t_c^*} - \frac{\alpha_g^* t_c^*}{\alpha_g t_c} \right) \frac{t_c \sigma_g^2 \cosh N\lambda_g}{2 \sinh N\lambda_g} \\ &+ \frac{\alpha_g^*}{2\sigma_g^{*2}} \left(\frac{\alpha_g t_c}{\alpha_g^* t_c^*} + \frac{\alpha_g^* t_c^*}{\alpha_g t_c} \right) D_{gck}^{(1)} + \frac{\alpha_g^*}{2\sigma_g^{*2}} \left(\frac{\alpha_g t_c}{\alpha_g^* t_c^*} - \frac{\alpha_g^* t_c^*}{\alpha_g t_c} \right) D_{gck}^{(2)} \\ &+ \frac{\alpha_g^*}{2\sigma_g^{*2}} \frac{\alpha_g^* t_c^*}{\alpha_g t_c} \left(\theta_{gk}^* - \theta_{gk} \right) \left(2 \frac{\cosh N\lambda_{gc} - 1}{\sinh N\lambda_{gc}} (X_{gc0} + X_{gcN} - 2\theta_{gk}) \right) \\ &- \frac{\alpha_g^{*2} t_c^*}{2\sigma_g^{*2}} (\theta_{gk}^* - \theta_{gk})^2 + \frac{\alpha_g^*}{2\sigma_g^{*2}} ((X_{gc0} - \theta_{gk}^*)^2 - (X_{gcN} - \theta_{gk}^*)^2) + \mathcal{O}(1/N), \end{aligned}$$

where

$$D_{gck}^{(1)} = -\frac{\cosh N\lambda_{gc}}{2\sinh N\lambda_{gc}}((X_{gc0}-\theta_{gk})^2 + (X_{gcN}-\theta_{gk})^2) + \frac{1}{\sinh N\lambda_{gc}}(X_{gc0}-\theta_{gk})(X_{gcN}-\theta_{gk})$$

$$D_{gck}^{(2)} = -\frac{N\lambda_{gc}}{2\sinh^2 N\lambda_{gc}}((X_{gc0}-\theta_{gk})^2 + (X_{gcN}-\theta_{gk})^2) + \frac{N\lambda_{gc}\cosh N\lambda_{gc}}{\sinh^2 N\lambda_{gc}}(X_{gc0}-\theta_{gk})(X_{gcN}-\theta_{gk}).$$

Thus, the complete Q function is

$$\mathcal{Q} = \mathrm{E}_{Z,X}[\ln P(X,Z,E|S,\Theta,T)] = \sum_c \sum_k \left(\gamma_{ck}\ln\pi_k + \sum_g \gamma_{ck}\mathcal{Q}_{gck}\right) + \mathcal{O}(1/N).$$

2.1 Parameter optimization

The optimization equation is derived by solving the differentiation of the Q function likewise the parameter optimization of single gene, cell, and lineage model.

2.1.1 Optimization of θ_{gk}

$$\theta_{gk}^* = \theta_{gk} + \frac{\sum_c \gamma_{ck} \frac{2}{\alpha_g(1+\mathrm{e}^{-\alpha_g t_c})} (X_{gkN} - \mathrm{e}^{-\alpha_g t_c} X_{gk0} - (1-\mathrm{e}^{-\alpha_g t_c})\theta_{gk})}{\sum_c \gamma_{ck} t_c}$$

2.1.2 Optimization of α_g

$$\alpha_g^* = \frac{\sum_c \sum_k \gamma_{ck}\left(-t_c\sigma_g^2 - (X_{gc0}-\theta_{gk})^2 + (X_{gcN}-\theta_{gk})^2\right)}{\left(\sum_c \sum_k \gamma_{ck} Z_{cgk}^{\alpha}\right)}$$

2.1.3 Optimization of σ_g^2

$$\sigma_g^{*2} = \frac{1}{C}\sum_c \sum_k \gamma_{gck} \frac{2\alpha_g}{1-\mathrm{e}^{-2\alpha_g t_c}} \left(X_{gkN} - \mathrm{e}^{-\alpha_g t_c} X_{gk0} - (1-\mathrm{e}^{-\alpha_g t_c})\theta_{gk}\right)^2$$

2.1.4 Optimization of t_c

We optimize t_c so that it satisfies following equation.

$$\sum_k \sum_g \gamma_{ck}\left(\alpha_g t_c \left(1 - \frac{\cosh \alpha_g t_c}{\sinh \alpha_g t_c}\right) - 2\frac{\alpha_g}{\sigma_g^2} D_{gck}^{(2)}\right) = 0$$

We used Newton's method as follows:

$$
\begin{aligned}
f(t_c) =& \sum_k \sum_g \gamma_{ck} \left(\alpha_g t_c \left(1 - \frac{\cosh \alpha_g t_c}{\sinh \alpha_g t_c} \right) - 2 \frac{\alpha_g}{\sigma_g^2} D_{gck}^{(2)} \right) \\
\frac{df(t_c)}{dt_c} =& \sum_k \sum_g \gamma_{ck} \Big(\alpha_g \left(1 - \frac{\cosh \alpha_g t_c}{\sinh \alpha_g t_c} \right) + \frac{\alpha_g^2 t_c}{\sinh^2 \alpha_g t_c} \\
& - 2 \frac{\alpha_g^2}{\sigma_g^2} \frac{1}{\sinh^3 \alpha_g t_c} \Big(- \frac{1}{2} (\sinh \alpha_g t_c - 2\alpha_g t_c \cosh \alpha_g t_c)((X_{cg0} - \theta_{gk})^2 + (X_{cgN} - \theta_{gk})^2) \\
& + (\cosh \alpha_g t_c \sinh \alpha_g t_c - \alpha_g t_c (1 + \cosh^2 \alpha_g t_c))(X_{cg0} - \theta_{gk})(X_{cgN} - \theta_{gk}) \Big) \Big) \\
t_{cn+1} =& \, t_{cn} - \frac{f(t_{cn})}{f'(t_{cn})}.
\end{aligned}
$$

2.1.5 Optimization of π_k

$$
\pi_k = \frac{\sum_c \gamma_{ck}}{\sum_c \sum_{k'} \gamma_{ck'}}
$$

2.2 Expected value of S_{cg}

Thus far, we assume S_{cg} (X_{cg0}) is given. However, it is unobserved practically and we have to calculate expected value of S_{gc}.

$$
\begin{aligned}
P(S_{cg}|E_{cg}) &= \frac{P(E_{cg}|S_{cg})P(S_{cg})}{P(E_{cg})} \\
&\propto \mathcal{N}\left(E_{cg}|\mathrm{e}^{-\alpha t} S_{cg} + (1 - \mathrm{e}^{-\alpha_g t_c})\theta_{gk}, V_{gc}\right) \mathcal{N}(S_{cg}|\mu_{0g}, \sigma_{0g}^2) \\
&\propto \mathcal{N}\left(S_{cg} \middle| \frac{V'^{-1}\mu' + \sigma_{0g}^{-2}\mu_{0g}}{V'^{-1} + \sigma_{0g}^{-2}}, \frac{1}{V'^{-1} + \sigma_{0g}^{-2}} \right),
\end{aligned}
$$

where

$$
\begin{aligned}
\mu' &= \mathrm{e}^{\alpha_g t_c} E_{cg} + (1 - \mathrm{e}^{\alpha_g t_c})\theta_{gk} \\
V' &= \mathrm{e}^{2\alpha_g t_c} V_{gc} \\
V_{gc} &= \frac{\sigma_g^2 (1 - \mathrm{e}^{-2\alpha_g t_c})}{2\alpha_g}.
\end{aligned}
$$

Therefore, the expected values related to X_{cg0} are given by

$$
\begin{aligned}
\mathrm{E}[X_{cg0}^2] &= \left(\frac{V'^{-1}\mu' + \sigma_{0g}^{-2}\mu_{0g}}{V'^{-1} + \sigma_{0g}^{-2}} \right)^2 + \frac{1}{V'^{-1} + \sigma_{0g}^{-2}} \\
\mathrm{E}[X_{cg0}] &= \frac{V'^{-1}\mu' + \sigma_{0g}^{-2}\mu_{0g}}{V'^{-1} + \sigma_{0g}^{-2}},
\end{aligned}
$$

and we just substitute the above $\mathrm{E}[X_{cg0}]$ and $\mathrm{E}[X_{cg0}^2]$ into X_{cg0} and X_{cg0}^2, respectively, for parameter optimization.

2.3 The marginal log-likelihood

S_{gc} is not observed and we have to marginalize over S_{gc} to calculate the marginal log-likelihood, and it is described as follows:

$$\int dS_{gc} N(E_{gc}|\mathrm{e}^{-\alpha t} S_{gc} + (1-\mathrm{e}^{-\alpha t})\theta_{gk}, V_g) N(S_{gc}|\mu_{0g}, \sigma_{0g}^2) = N(E_{gc}|\mathrm{e}^{-\alpha t}\mu_{0g} + (1-\mathrm{e}^{-\alpha t})\theta_{gk}, V + \mathrm{e}^{-2\alpha t}\sigma_{0g}^2).$$

Therefore, the log-likelihood of E is

$$l(E) = \sum_c \log\left(\sum_k \pi_k \prod_g \mathcal{N}\left(E_{cg}|\mathrm{e}^{-\alpha_g t_c}\mu_{0g} + (1-\mathrm{e}^{-\alpha_g t_c})\theta_{gk}, V_g + \mathrm{e}^{-2\alpha_g t_c}\sigma_{0g}^2\right)\right).$$

3 A procedure of parameter optimization

In this research, we used following parameter optimization procedure to avoid sub-optimal solutions.

Firstly, we initialized pseudo-time t_c based on dimension reduction approach and $\alpha_g, \sigma_g^2, \theta_g$ by using the model of $K = 1$.

1. Add the mean of initial distribution ($\mu_0 = \{\mu_{0g}|g = 0, ..., G\}$) as a root to single-cell expression data, and perform principal component analysis.
2. Calculate minimum spanning tree with Prim's algorithm on D dimensional latent space and calculate the shortest path from root (μ_0) to a cell c and define the standardized of the total weight of the shortest path as the initial value of t_c (In this research, we set $D = 2$ unless we refer).
3. Set the initial value of θ_g to the mean of the expression ($\theta_g = \frac{1}{C}\sum_{c=1}^{C} E_{gc}$).
4. Initialize α_g, σ_g^2 (In this research, we set $\alpha_g = 5.0, \sigma_g^2 = 1.0$).
5. Optimize $\alpha_g, \sigma_g^2, \theta_g$ with SCOUP of $K = 1$ by EM algorithm with 10 iterations.

Secondary, we optimize parameter of mixture model. Because parameters fell into a sub-optimal solution which shows wrong order of cells when we optimized all parameter simultaneously, we optimized parameters except t_c at first.

1. Initialize θ_{gk} with θ_g calculated by above procedure.
2. Initialize the expectation of a latent value of a cell (γ_{ck}) randomly and calculate other statistic, and optimize α_g, σ_g^2, and θ_{gk} with M-step.
3. Run E-step to calculate γ_{ck} and other statistic.
4. Run M-step to optimize α_g, σ_g^2, and θ_{gk}.
5. Return to step 3 until the number of iterations reach m_1 (In this research, we set $m_1 = 1,000$.).

Lastly, we optimize all parameters.

1. Run E-step

2. Run M-step. We optimize t_c at first, and optimized other parameters after that.

3. Stop the parameter optimization if $|\mathrm{e}^{-\alpha_g^* t_{\max}}\theta_{gk}^* - \mathrm{e}^{-\alpha_g t_{\max}}\theta_{gk}|$, $|\frac{\sigma_g^{*2}(1-\mathrm{e}^{-2\alpha_g^* t_{\max}})}{2\alpha_g^*} - \frac{\sigma_g^2(1-\mathrm{e}^{-2\alpha_g t_{\max}})}{2\alpha_g}|$, and $|t_c^* - t_c|$ are under ϵ (In this research, we set $\epsilon = 0.01$). We used these values to check convergence because these are meaningful values and α_g and σ_g^2 can change together so that the likelihood does not change (see next section).

4. Stop the parameter optimization if the number of iterations reach m_2 (In this research, we set $m_2 = 10,000$ and verified that parameters are converged before m_2 iterations under most conditions).

5. Return to step 1.

α_g can be very large and small which is meaningless to estimate accurately and we set lower and upper bounds to $\alpha_{\min} = 0.1, \alpha_{\max} = 100$ and set $\alpha_g = \alpha_{\min}$ ($\alpha_{\max}$) if α_g^* is under (over) $\alpha_{\min}$ ($\alpha_{\max}$). σ_g^2 can be significantly small and we set the lower bound similar way ($\sigma_{\min}^2 = 0.1$). We also set the bounds of pseudo-time so that the lower bound is $t_{\min} = 0.001$ and the upper bound is $t_{\max} = 2.0$. For pseudo-time (t_c) optimization, we stop the Newton's method if $|t_{cn+1} - t_{cn}|$ is lower than ϵ or the number of iteration reach 100. The solution of Newton's method t_c can be incorrect value and we set the new parameter of t_c to the time whose likelihoods are highest in the three case: t_c is old time; t_c is the solution of Newton's method; $t_c = t_{\min}$; and $t_c = t_{\max}$.

4 Validation of parameter optimization method

In this section, we validated the parameter optimization method with simulation data. We generated simulation data with $C = 100$, $G = 500$, and $K = 1$, and each parameter was sampled so that $t_c = U_R[0,1]$, $\alpha_g = U_R[0.1, 10]$, $\sigma_g^2 = U_R[0.1, 100]$, and $\theta_g = U_R[-5, 5]$, where $U_R[a, b]$ is a uniform random number from a to b. All of the initial distributions of the gene were $N(X_{cg0}|0, 1.0)$ and X_{cg0} was sampled from the distribution. To complete the scale of the parameters, we set $t_{max} = 1.0$. With above conditions, we sampled the expression data from the normal distribution based on OU process, and applied this simulation data for SCOUP.

Firstly, we compared the values of estimated parameters with those of true parameters (Figure 1(A,B,C,D)). The values of estimated time is highly correlated with true values ($r^2 = 0.94$). The difference between estimated time and true time becomes large for large t_c. This is because the distribution of OU-process becomes stationary distribution for t sufficiently large, and the fluctuation of the value of optimized t_c will be large for such condition. The values of estimated θ_g is also highly correlated with true values ($r^2 = 0.96$). But estimated θ_g of a few genes are different from true values significantly. This will be because the influence of θ_g on the distribution is significantly small when $\alpha_g \simeq 0$, and hence, the value of θ_g is unstable in such condition. The values of estimated α_g and σ_g^2 are highly correlated with true values (r^2 is 0.79 and 0.77, respectively), but estimated α_g and σ_g^2 of some genes are significantly larger than true values. This is because that the distribution of different α_g and σ_g^2 will be almost equal for the gene of $\theta_g \simeq \mu_{0g}$ as long as the balance between α_g and σ_g^2 are kept, and the estimated absolute values will be unstable. Then, we investigated the value of mean ($\mathrm{e}^{-\alpha_g t}\theta_g$) and variance ($\sigma_g^2(1 - \mathrm{e}^{-2\alpha_g t})/2\alpha_g$) of OU process at time $t = 1$ (Figure 1(E,F)). The values of estimated mean and variance are highly correlated with those of true mean and variance (0.99 and 0.94, respectively), and hence, SCOUP succeed to reconstruct the original probabilistic distribution with high accuracy.

Next, we investigated that the log-likelihood of optimized parameters is higher than those of varied parameters. Figure 2 is the example of the log-likelihood curve with respect to time parameter of a cell (t_c), and the value of optimized t_c is drawn with x-mark. The log-likelihood of the optimized t_c is located in the top of the log-likelihood curve. As shown in Figure 2(C), the log-likelihood are almost equal $0.5 < t_c$ because the distribution will almost be equal to the stationary distribution for large t_c. Figure 3 is the example of the log-likelihood surface with respect to α_g and θ_g of a gene. The log-likelihood of the optimized α_g and θ_g are located in the top of the log-likelihood surface. Figure 4 is also the example of the log-likelihood surface with respect to σ_g^2 and θ_g of a gene. The log-likelihood of the optimized σ_g^2 and θ_g are located in the top of the log-likelihood surface. Figure 5 is also the example of the log-likelihood surface with respect to θ_g and σ_g^2 of a gene. Although the log-likelihood of the optimized α_g and σ_g^2 are located in the top of the log-likelihood surface, the log-likelihood are almost equal for $\sigma_g^2 \simeq 20 \times \alpha_g$ regarding a gene (Figure 5(C)). This is because that the distribution of different α_g and σ_g^2 will be almost equal in some conditions as mentioned in the above paragraph.

Although the values of optimized parameters have potential to be unstable in some conditions, the mean and variance of OU process are stable and the log-likelihood of optimized parameters are highest. Therefore, we conclude that SCOUP succeed to optimize parameters.

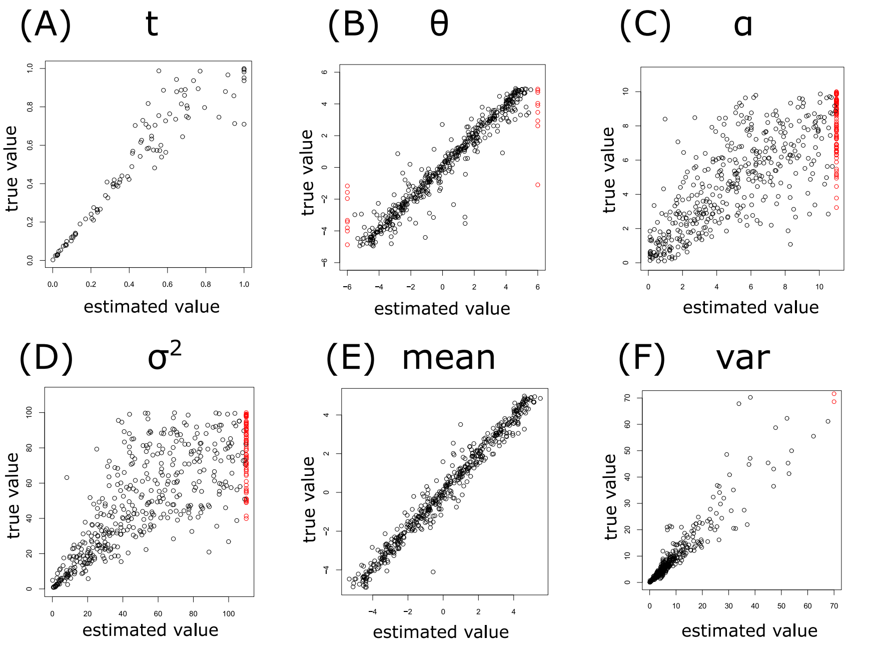

Figure 1: Comparison between the estimated values and true values: (A) for pseudo-time (t), (B) for θ_g, (C) for α_g, (D) for σ_g^2, (E) for mean, (F) for variance. The outlier whose estimated value exceeds the boundary of drawing area is visualized in the border with a red circle for visualization.

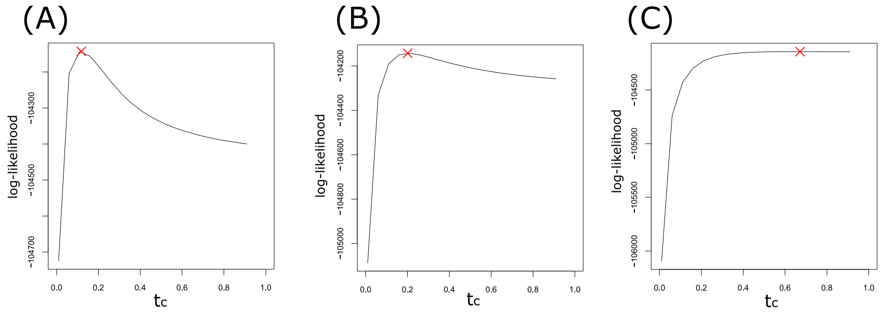

Figure 2: The log-likelihood curve with respect to t_c of a cell. The optimized t_c is indicated with x-max.

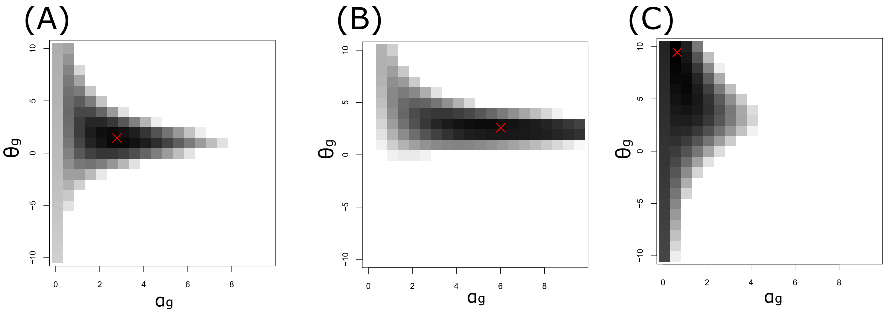

Figure 3: The log-likelihood surface with respect to α_g and θ_g of a gene. The color of a pixel represents the log-likelihood and black represents the highest log-likelihood. The optimized (α_g, θ_g) is indicated with x-max.

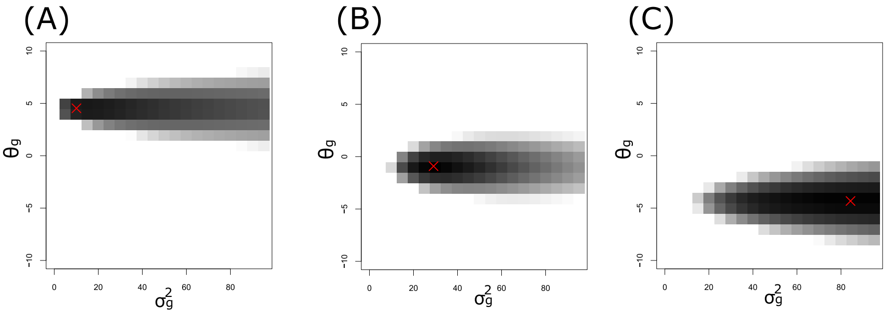

Figure 4: The log-likelihood surface with respect to σ_g^2 and θ_g of a gene. The color of a pixel represents the log-likelihood and black represents the highest log-likelihood. The optimized (σ_g^2, θ_g) is indicated with x-max.

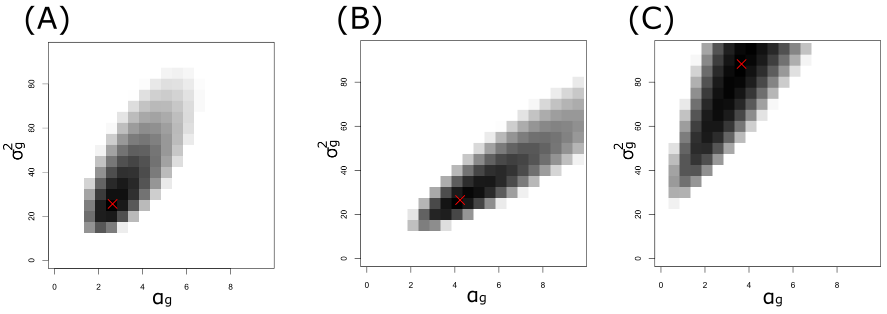

Figure 5: The log-likelihood surface with respect to α_g and σ_g^2 of a gene. The color of a pixel represents the log-likelihood and black represents the highest log-likelihood. The optimized (α_g, σ_g^2) is indicated with x-max.

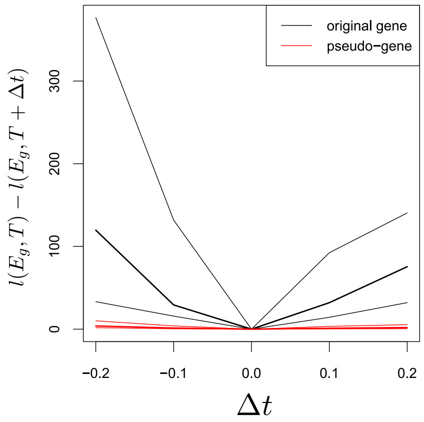

Figure 6: $l(E_g, T) - l(E_g, T + \Delta t)$ for normal gene (black) and pseudo-gene (red) for various Δt. The bold and narrow lines indicate median and quantile, respectively.

Table 1: The runtimes for original Kouno's data, Moignard's data (1,000 cells), and Shalek's data (LPS).

	Kouno's data	Moignard's data	Shalek's data
runtimes (min)	8	26	91

5 Automatic reduction of unrelated gene in pseudo-time estimation

The expression data of gene, which is unrelated to differentiation, can fluctuate the pseudo-time estimation in the previous methods. In contrast, SCOUP estimates pseudo-time while automatically reducing the influence of unrelated gene. This is because the probabilistic distribution of such unrelated gene will be constant in relation to time parameter and such gene will not affect optimization of time parameter.

In this section, we verified that the unrelated genes actually do not affect pseudo-time estimation of SCOUP. We defined the gene log-likelihood for various time as follows:

$$l(E_g, T + \Delta t) = \sum_c \log \left(\mathcal{N} \left(E_{cg} | \mathrm{e}^{-\alpha_g (t_c + \Delta t)} \mu_{0g} + (1 - \mathrm{e}^{-\alpha_g (t_c + \Delta t)}) \theta_{gk}, V_g + \mathrm{e}^{-2\alpha_g (t_c + \Delta t)} \sigma_{0g}^2 \right) \right),$$

where each parameter is optimized and Δt is the fluctuation of time. However, $(t_c - \Delta t)$ is set to the boundary value if it exceeds $t_{\min}$ or $t_{\max}$.

We used original Kouno's data with 45 pseudo-gene for validation. The expression of pseudo-gene were constructed by shuffling raw expression data among 960 cells for each gene. Figure 6 shows the median and quartile of $l(E_g, T) - l(E_g, T + \Delta t)$ for original gene and pseudo-gene for various Δt. In contrast to the original gene, whose gene log-likelihood is maximum at $\Delta t = 0.0$, the gene log-likelihood of pseudo-gene are approximately constant. Thus, the pseudo-gene does not affect the pseudo-time optimization because the gene log-likelihood is not affected by the time parameter.

6 Computational complexity

The computational complexity of SCOUP is $\mathcal{O}(IKCG)$, where I is the number of optimization iterations. Therefore, the runtime of SCOUP increases linearly with the number of cells and genes. The runtimes for original Kouno's data, Moignard's data (1,000 cells), and Shalek's data (LPS) about 8 min, 26 min, and 91 min, respectively (Table 1). The computations were performed on my MacBookPro equipped with 2.8 GHz Intel Core i5 processors and 16 GB RAM. Thus, SCOUP works well with short runtimes with laptop computers.

7 Estimation of the number of lineages (K)

We investigated whether SCOUP could estimate the number of lineages (K) in the case that the genuine number of lineage was unknown. We applied SCOUP for Kouno's data(2) ($\epsilon = 1.0$) in the condition that K were set to either 1, 2, 3, or 4.

Figure 7A shows the box plot of the log-likelihood for each condition ($K = 1, 2, 3, 4$). In comparison with the difference between the log-likelihood of $K = 1$ and that of $K = 2$, the log-likelihood of $K = 2, 3, 4$ are

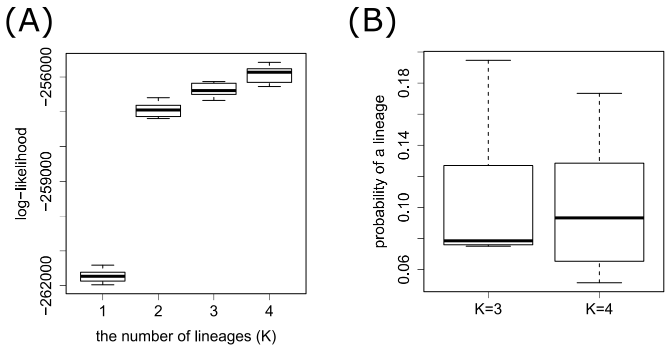

Figure 7: (A) The box plot of the log-likelihood for various K. (B) The box plot of the probability of the remaining lineage (π_k) after removing two dominant lineage for $K = 3, 4$ model.

close. Therefore, the model with $K = 2$ is approximately sufficient to describe the data. In addition, the probability of a lineage k (π_k) can be used for K estimation. Figure 7B is the box plot of the remaining π_k after removing two dominant lineage for $K = 3, 4$ model. In both conditions, many π_k are under 0.1. Thus, the probability of a lineage is fitted to relatively small when K is set to more than the genuine number of lineages, and we can infer the appropriate number of lineages.

8 Cell lineage estimation with Gaussian mixture model

We estimated cell lineage with Gaussian mixture model (GMM) implemented in mclust package [2]. The AUC values of mclust for Kouno's data(2) ($\epsilon = 0.0$) and Moignard's data are 0.86 and 0.96, respectively, and both of them are inferior to those of SCOUP (0.99 and 1.0). Figure 8 and 9 show cells of Kouno's data and Moignard's data in the space of first two PCs and the colors of cells indicate the genuine cell lineage (left), the lineage estimated using SCOUP (middle), and the lineage using mclust (right). GMM cannot estimate cell lineage correctly for cells at an early stage of bifurcation because GMM does not count the time axis and will work well only for cells whose expression pattern changes sufficiently after bifurcation. Moreover, mclust seems to overfit the 4SG cells around (-6, 0) in the PCA space, and failed to distinguish a portion of 4SG cells for Moignard's data (Figure 9). This is because GMM will fit to the position in which large number of cells exist, and GMM cannot estimate the path of bifurcation in the condition that cells are unevenly distributed . Therefore, it is important to count time axis to analyze expression for cells during bifurcation.

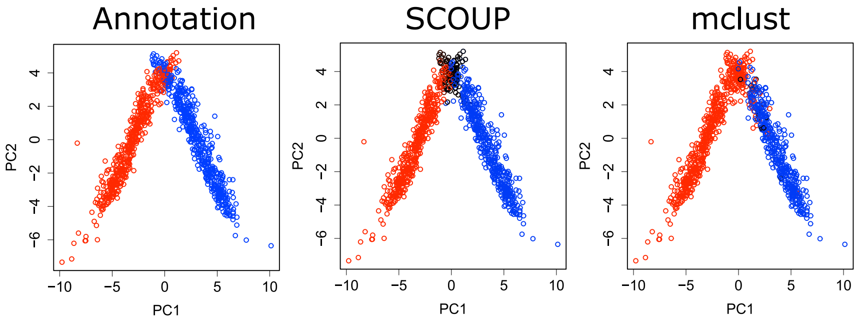

Figure 8: PCA of cells of Kouno's data based on gene expression. The cell colors indicate the genuine lineage (left), lineage estimated with SCOUP (middle), and lineage estimated with mclust (right). The color for SCOUP is defined by γ_{c0}; black, 0.5; red, 0.0; and blue, 1.0. The color for mclust is defined by expectation of latent values; black, 0.5; red, 0.0; and blue, 1.0. The color of each state is consist among plots.

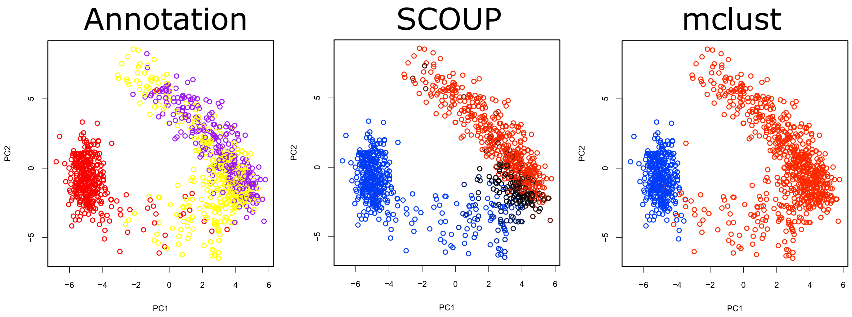

Figure 9: PCA of cells of Moignard's data based on gene expression. The cell colors represent the genuine lineage (left), lineage estimated with SCOUP (middle), and lineage estimated with mclust (right). The color for the genuine lineage is defined by the annotation of the cell; yellow, HF; red, 4SG; and purple, $4SFG^-$. The color for the SCOUP analysis is defined by γ_{c0}; black, 0.5; red, 0.0; and blue, 1.0. The color for the mclust analysis is defined by expectation of latent values; black, 0.5; red, 0.0; and blue, 1.0. We determined the color of each state so that they consist among each plot.

9 Annotated pairs in the top 1,000 $C_{\mathbf{Raw}}$ and $C_{\mathbf{Std}}$ values

As described in the main manuscript, we investigated whether the target genes of a transcription factor (TF) can be predicted under the assumption that the expression of a TF and its target genes are highly correlated. The top 1,000 C_{Raw} and C_{Std} values contained correlations of There are 24 and 27 annotated pairs in the top 1,000 C_{Raw} and C_{Std} values, respectively (Table 2). Only three annotated pairs (UHRF1, RRM2), (MCM5, RRM2), and (MCM4, RRM2), were common between the 24 C_{Raw} correlation values and the 27 C_{Std} correlation values.

Table 2: The annotated pairs in the top 1,000 C_{Raw} and C_{Std} values. The left and right tables correspond to C_{Raw} and C_{Std}, respectively. The first column of each table contains the TF names and the second column lists the target gene names. The third column contains the C_{Raw} or C_{Std} of the pairs.

TF	target gene	C_{Raw}	TF	target gene	C_{Std}
IFIT1	RTP4	0.761	UHRF1	RRM2	0.666
IFIT1	IFI47	0.760	MCM5	RRM2	0.644
IFIT1	OASL2	0.746	MCM4	RRM2	0.557
IFI205	IFI47	0.702	MCM5	CDCA8	0.489
IRF7	IFI47	0.699	ATAD2	RRM2	0.486
IRF7	OASL2	0.694	MCM3	RRM2	0.482
UHRF1	RRM2	0.681	UHRF1	CDCA8	0.480
IFI205	RTP4	0.681	CENPA	TOP2A	0.476
IFIT3	MPA2L	0.681	MCM5	TOP2A	0.470
IFIT1	USP18	0.680	MCM3	2810417H13RIK	0.464
IFI205	PYHIN1	0.658	HMGB2	TOP2A	0.447
IFI203	RTP4	0.655	MCM3	TOP2A	0.447
MCM5	RRM2	0.653	PLD4	FCGR2B	0.445
IRF7	USP18	0.651	MCM5	MAD2L1	0.437
PARP14	IFI47	0.649	H2AFZ	2810417H13RIK	0.434
IFIH1	IFI47	0.645	MCM4	TOP2A	0.432
IFIH1	RTP4	0.641	ATAD2	2810417H13RIK	0.422
IFIH1	OASL2	0.637	MCM4	CDCA8	0.421
IFIT1	IGTP	0.619	MCM5	DNAJC9	0.421
IFI205	MPA2L	0.605	ATAD2	TOP2A	0.413
IRF7	RTP4	0.593	MCM5	DTYMK	0.408
IFI205	GBP2	0.592	MCM4	DTYMK	0.408
PARP9	USP18	0.587	H2AFZ	TOP2A	0.404
MCM4	RRM2	0.584	MCM4	ANP32E	0.402
			MCM4	LBR	0.402
			UHRF1	CKS1B	0.387
			MCM3	ANP32E	0.383

References

[1] Hu, G.Y., O'Connell, R.F.: Analytical inversion of symmetric tridiagonal matrices. J.Phys.A **29**(7), 1511–1513 (1996)

[2] Fraley, C., Raftery, A.E., Murphy, T.B., Scrucca, L.: mclust Version 4 for R: Normal Mixture Modeling for Model-Based Clustering, Classification, and Density Estimation. Technical Report **597** (2012)
